# BAF155 methylation drives metastasis by hijacking super-enhancers and subverting anti-tumor immunity

**DOI:** 10.1093/nar/gkab1122

**Published:** 2021-11-19

**Authors:** Eui-Jun Kim, Peng Liu, Shengjie Zhang, Kristine Donahue, Yidan Wang, Jennifer L Schehr, Serena K Wolfe, Amber Dickerson, Li Lu, Lixin Rui, Xuehua Zhong, Kari B Wisinski, Min Yu, Aussie Suzuki, Joshua M Lang, Irene M Ong, Wei Xu

**Affiliations:** McArdle Laboratory for Cancer Research, University of Wisconsin-Madison, Madison, WI 53706, USA; Carbone Comprehensive Cancer Center, University of Wisconsin, Madison, WI 53706, USA; Department of Biostatistics and Medical Informatics. School of Medicine and Public Health, University of Wisconsin, Madison, WI 53706, USA; Carbone Comprehensive Cancer Center, University of Wisconsin, Madison, WI 53706, USA; McArdle Laboratory for Cancer Research, University of Wisconsin-Madison, Madison, WI 53706, USA; McArdle Laboratory for Cancer Research, University of Wisconsin-Madison, Madison, WI 53706, USA; Carbone Comprehensive Cancer Center, University of Wisconsin, Madison, WI 53706, USA; McArdle Laboratory for Cancer Research, University of Wisconsin-Madison, Madison, WI 53706, USA; Carbone Comprehensive Cancer Center, University of Wisconsin, Madison, WI 53706, USA; Department of Medicine, University of Wisconsin, Madison, WI 53706, USA; Carbone Comprehensive Cancer Center, University of Wisconsin, Madison, WI 53706, USA; Department of Medicine, University of Wisconsin, Madison, WI 53706, USA; Carbone Comprehensive Cancer Center, University of Wisconsin, Madison, WI 53706, USA; Department of Stem Cell Biology and Regenerative Medicine, and USC Norris Comprehensive Cancer Center, Keck School of Medicine, University of Southern California, Los Angeles, CA, USA; Wisconsin Institute for Discovery, University of Wisconsin-Madison, Madison, WI 53715, USA; Laboratory of Genetics, University of Wisconsin-Madison, Madison WI, USA; Department of Obstetrics and Gynecology, School of Medicine and Public Health, University of Wisconsin, Madison, WI 53706, USA; Carbone Comprehensive Cancer Center, University of Wisconsin, Madison, WI 53706, USA; Wisconsin Institute for Discovery, University of Wisconsin-Madison, Madison, WI 53715, USA; Laboratory of Genetics, University of Wisconsin-Madison, Madison WI, USA; Department of Medicine, University of Wisconsin, Madison, WI 53706, USA; Carbone Comprehensive Cancer Center, University of Wisconsin, Madison, WI 53706, USA; Department of Stem Cell Biology and Regenerative Medicine, and USC Norris Comprehensive Cancer Center, Keck School of Medicine, University of Southern California, Los Angeles, CA, USA; McArdle Laboratory for Cancer Research, University of Wisconsin-Madison, Madison, WI 53706, USA; Carbone Comprehensive Cancer Center, University of Wisconsin, Madison, WI 53706, USA; Department of Medicine, University of Wisconsin, Madison, WI 53706, USA; Carbone Comprehensive Cancer Center, University of Wisconsin, Madison, WI 53706, USA; Department of Obstetrics and Gynecology, School of Medicine and Public Health, University of Wisconsin, Madison, WI 53706, USA; Department of Biostatistics and Medical Informatics. School of Medicine and Public Health, University of Wisconsin, Madison, WI 53706, USA; Carbone Comprehensive Cancer Center, University of Wisconsin, Madison, WI 53706, USA; McArdle Laboratory for Cancer Research, University of Wisconsin-Madison, Madison, WI 53706, USA; Carbone Comprehensive Cancer Center, University of Wisconsin, Madison, WI 53706, USA

## Abstract

Subunits of the chromatin remodeler SWI/SNF are the most frequently disrupted genes in cancer. However, how post-translational modifications (PTM) of SWI/SNF subunits elicit epigenetic dysfunction remains unknown. Arginine-methylation of BAF155 by coactivator-associated arginine methyltransferase 1 (CARM1) promotes triple-negative breast cancer (TNBC) metastasis. Herein, we discovered the dual roles of methylated-BAF155 (me-BAF155) in promoting tumor metastasis: activation of super-enhancer-addicted oncogenes by recruiting BRD4, and repression of interferon α/γ pathway genes to suppress host immune response. Pharmacological inhibition of CARM1 and BAF155 methylation not only abrogated the expression of an array of oncogenes, but also boosted host immune responses by enhancing the activity and tumor infiltration of cytotoxic T cells. Moreover, strong me-BAF155 staining was detected in circulating tumor cells from metastatic cancer patients. Despite low cytotoxicity, CARM1 inhibitors strongly inhibited TNBC cell migration *in vitro*, and growth and metastasis *in vivo*. These findings illustrate a unique mechanism of arginine methylation of a SWI/SNF subunit that drives epigenetic dysregulation, and establishes me-BAF155 as a therapeutic target to enhance immunotherapy efficacy.

## INTRODUCTION

Protein arginine methylation is an abundant post-translational modification catalyzed by nine mammalian arginine methyltransferases (PRMTs). PRMT4, also known as coactivator-associated arginine methyltransferase 1 (CARM1), asymmetrically di-methylates protein substrates on arginine residues (1). CARM1 is indispensable in mammals. Both CARM1 knock-out mice and enzyme-inactive CARM1 knock-in mice die at birth and display identical developmental defects, underscoring its essential role in physiology (2,3). Emerging evidence supports an oncogenic function of CARM1 in human cancer. CARM1 is overexpressed in a variety of cancer types, and its high expression correlates with poor prognosis, particularly in triple-negative breast cancer (TNBC), which lacks expression of ER, PR and HER2 (4,5). Overexpression of CARM1 induced hyper-branching of the mammary glands, increased Ki-67 staining, and augmented HER2-oncogene-induced tumor formation in mouse models (6). CARM1 functions as both a coactivator and a methyltransferase (7). CARM1 interacts with cancer-relevant transcription factors (e.g. NF-κB, p53, E2F1) and co-activators (e.g. CBP/p300), to modulate gene expression and promote cancer progression. In addition to the established role of histone H3 methylation in transcriptional activation of estrogen receptor (ER) target genes (8), methylation of non-histone substrates regulates distinct hallmarks of cancer (9). For example, BAF155 methylation drives cancer metastasis (10), providing the foundation for employing CARM1 inhibitors in cancer therapy. Global profiling of CARM1 substrates revealed that chromatin regulators and RNA processing proteins are CARM1 substrates (11), uncovering novel cancer susceptibilities and therapeutic vulnerabilities to CARM1 inhibition. Indeed, several CARM1-specific inhibitors have been developed and tested in preclinical models. TP-064 and EZM2302 elicited anti-proliferative effects on leukemia and multiple myeloma cells (12–14), and SKI-73 showed anti-migratory effects in breast cancer cells (15).

Super-enhancers (SEs) are characterized as a large stretch of enhancers spanning several kilobases. SEs are occupied at a high density by transcription factors (TFs), co-activators (e.g. MED1), and RNA polymerase II (Pol II), and determine cell identity (16). SEs are enriched with active histone marks, such as H3K4me1 and H3K27Ac, the latter of which recruits bromodomain and extra-terminal domain (BET) proteins. Coactivators (e.g. BRD4) form phase-separated condensates at SEs to compartmentalize and concentrate transcriptional machinery at key cell-identity genes (17). Intriguingly, cancer cells also hijack large SEs to regulate the expression of key oncogenes (18), suggesting that cancer cells are addicted to a SE-driven transcriptional program, which is a therapeutic vulnerability. Pharmacological inhibition of BRD4, a member of the BET protein family, by BET inhibitors (BETi), is a well-established example of SE component perturbation (18). Although BETi elicits strong anti-cancer effects in various cancer types, and some inhibitors have advanced to human clinical trials, these drugs are often associated with adverse side-effects because they also target non-cancer cells (e.g. hematopoietic stem cells) (19).

In this study, we identified dual roles for me-BAF155 in promoting tumor metastasis: activation of oncogenes and repression of immune response in TNBC. First, global mapping of methylated BAF155 (me-BAF155) genomic binding sites using chromatin immunoprecipitation sequencing (ChIP-seq) revealed co-localization of me-BAF155 and BRD4 at SEs to regulate oncogene expression. CARM1 inhibitor (CARM1i) treatment reduced the number of SEs by over 80% as well as global BRD4 binding, ablating SE-addicted oncogene expression. Although both CARM1i and JQ1 reduced global BRD4 association, CARM1i EZM2302 inhibited, whereas JQ1 promoted, growth and metastasis in a 4T1.2 syngeneic mouse model. Second, CARM1i treatment strongly activated interferon-stimulated genes (ISGs), and enhanced CD8^+^ T cell activity and tumoral infiltration. Bioinformatic analyses of me-BAF155 binding sites near ISGs identified a conserved binding motif of B-cell lymphoma 11A (BCL11A), a transcription factor and a component of the SWI/SNF complex. Further studies demonstrated that ISGs were repressed by me-BAF155 and HDAC1, but activated by BCL11A and PBAF. Pharmacological inhibition of CARM1 not only abrogated oncogene addiction to SEs, but also boosted immune response, both of which depend on ablation of BAF155 methylation. The dual function of me-BAF155 in driving cancer metastasis provides a scientific basis for developing epigenetic therapies for TNBC.

## MATERIALS AND METHODS

### Animal models

All mice were maintained in accordance with protocols approved by the Research Animal Resource Center of University at the Wisconsin-Madison and the study was compliant with ethical regulations regarding animal research. Five to six week-old female NOD/SCID mice were used for the patient derived xenograft experiments. Balb/c mice were used for the 4T1.2 injection model. HCI-002 and HCI-009 PDX models (26) were generous gifts from Dr. Alana Welm (University of Utah). The 4T1.2 cell line (model) was a generous gift from Dr. Yibin Kang (Princeton University). Patient consent for tumor implantation in mice was obtained under protocols approved by the IRB (IRB00050634). HCI-002 and HCI-009 tumor tissues ∼2–5 mm^3^ in size were minced and passed through an 18 G and 20 G needle in PBS to produce a cell suspension. The suspension was subcutaneously injected into the mammary fat pads of the mice, and allowed to form visible tumors. When the tumors reached ∼50 mm^3^ in size, after ∼3–4 weeks, mice were randomized to treatment with the following compounds: 50 mg/kg JQ1 in 10% hydroxypropyl beta cyclodextrin (Fisher, Pittsburgh, PA) by IP injection daily, or 50 mg/kg EZM2302 in 0.5% methylcellulose in water (Sigma-Aldrich, St. Louis, MO) by oral gavage twice a day. For the 4T1.2 injection model, 5 × 10^4^ cells were subcutaneously injected into the mammary fat pads of the mice. JQ1, EZM2302 or anti-CD8 monoclonal antibody (25 or 100 μg per mice) were treated or pre-treated for 3 days, as described in Figure 5A, H and Supplementary Figure S5A. Tumor size was measured every other day using calipers, and tumor volume was calculated using the following formula: *V* = (4/3) × π × (*L*/2) × (*L*/2) × (*D*/2). Luciferase-based noninvasive bioluminescent imaging and analysis were performed using the IVIS Imaging System (Caliper Life Sciences, Hopkinton, MA). 20 mg/ml d-luciferin was administered by IP injection, and mice were allowed to rest for 10 min prior to imaging. To compare variable initial tumor volumes, normalized tumor volume was calculated. Animals were sacrificed 14 days (HCI-002, HCI-009) or 28–30 days (4T1.2) after starting drug treatment.

### Cell culture and generation of BAF155 knockout cells

MCF-7, MDA-MB-231, MDA-MB-468, and HEK293T cells were purchased from ATCC and maintained in DMEM (Gibco, Carlsbad, CA) supplemented with 10% fetal bovine serum (FBS, HyClone, Logan, UT). Cells were incubated at 37°C with 5% CO_2_ in a humidified incubator. A guide RNA targeting BAF155 was inserted into the PX458 plasmid (Addgene #48138) to generate BAF155-KO cells using CRISPR-cas9. Guide sequence targeting human BAF155: 1# GCCTAGCTGTTTATCGACGGA; 2# AGCTGGATTCGGTGCGGGTC.

### Viral packaging and generation of stable cell lines

Lentiviral and retroviral packaging were performed as previously described (10). In brief, for lentiviral packaging, three plasmids (PAX2-, VSVG- and either pLKO.1-puro, or pxy-puro) were transfected into HEK293T cells and incubated for 48 h. For retroviral packaging, three plasmids (pHIT60-, VSVG-, and either pLNCX-BAF155 wild type, R1064K or R1064A plasmid) were used. pLKO.1-puro BCL11A was purchased from Sigma-Aldrich. pxy-puro BCL11A was a generous gift from Dr. Gregory Ippolito (Univ. of Texas at Austin). For adenoviral packaging, PacI-digested H2B-RFP (New England Biolabs, Ipswich, MA) was transfected into HEK293T cells and incubated for 48 hours. H2B-RFP plasmid was from Dr. Aussie Suzuki (Univ. of Wisconsin-Madison). To generate stable cell lines, 5 × 10^5^ cells were seeded in a 60 mm dish one day prior to virus infection. Subsequently, cells were incubated with 1 ml of pre-generated viral mixture and 1 ml of fresh medium which contained 10 μg/ml polybrene, followed by selection with 2 μg/ml puromycin for two weeks.

### Immunoblotting

Cells were lysed in RIPA buffer (Thermo Fisher Scientific, Waltham, MA) supplemented with the following protease inhibitors: 1 mM phenylmethylsulfonyl fluoride, 10 μg/ml aprotinin, 1 μM leupeptin, 10 μg/ml pepstatin, 1 mM sodium orthovanadate, and 1 mM sodium fluoride. The cells were lysed and sonicated using a Bioruptor (Diagenode, Denville, NJ) for 150 s. The concentrations of whole lysates was quantified using a Bradford assay. 50 μg of cell lysates was boiled in 5× Laemmli sample buffer, run in 6%, 10%, or 15% SDS-PAGE gels, and transferred to nitrocellulose membranes (VWR, Radnor, PA). Blots were blocked with 5% nonfat milk in T-TBS (20 mM Tris–Cl, pH 7.5, 150 mM NaCl and 0.1% Tween 20) for 1 h, and then incubated with primary antibodies at 4°C overnight. After three washes in T-TBS, blots were incubated with a peroxidase-labeled secondary antibody for 2 h at room temperature. After incubation, blots were washed three times in T-TBS and incubated with SuperSignal West Pico ECL (Thermo Fisher Scientific, Waltham, MA) before exposing to X-ray film. The following antibodies were used for immunoblotting: anti-me-BAF155, anti-me-PABP1, anti-PABP1 (10), anti-BAF155 (D7F8S, Cell Signaling Technology), anti-BRD4 (E2A7X, Cell Signaling Technology), anti-ARID1A (D2A8U, Cell Signaling Technology), anti-ARID1B (E9J4T, Cell Signaling Technology), anti-ARID2 (D8D8U, Cell Signaling Technology), anti-PBRM1 (D3F7O, Cell Signaling Technology), anti-BRG1 (D1Q7F, Cell Signaling Technology), anti-BCL11A (D4E3P, Cell Signaling Technology), anti-HDAC1 (D5C6U, Cell Signaling Technology), anti-IRF1 (D5E4, Cell Signaling Technology), anti-EGFR (Millipore), anti-phospho-BRD4 (Millipore), anti-TRPS1 (Bethyl), anti-SLITRK6 (GeneTex), anti-MMP7 (SantaCruz), anti-HIF-1α (H1alpha67, SantaCruz), anti-c-Myc (9E10, SantaCruz), MMP20 (Thermo Fisher Scientific), anti-STAT1 (15H3, Thermo Fisher Scientific), anti-STAT2 (1H10L19, Thermo Fisher Scientific), anti-ECOP (VWR), anti-EHF, anti-BRD7 (Abcam), anti-BRD9 (Abcam), anti-β-Actin (AC15, Sigma).

### Immunoprecipitation

Cells were lysed in hypotonic buffer (10 mM Tris pH 8.0, 1.5 mM magnesium chloride, 10 mM potassium chloride and protease inhibitors) and incubated at 4°C for 15 min. After incubation, 1/20 volume of detergent (10% triton X-100) was added to each sample, which were then vortexed and centrifuged at 13,000 g for 30 s. Hypertonic buffer (20 mM Tris pH 8.0, 1.5 mM magnesium chloride, 0.42 M potassium chloride, 20% glycerol and protease inhibitors) was added to the collected pellets (nuclear fraction) and supernatants (cytoplasmic fraction) and incubated at 4°C for 30 min with votexing every 10 min. After centrifugation at 13,000 g for 10 min, supernatants were transferred into 4× hypotonic buffer, mixed thoroughly, and centrifuged at 13,000 g for 10 min. Subsequently, the supernatants were collected and incubated at 4°C on a rotator with the following antibodies: anti-me-BAF155 (10), anti-BAF155 (D7F8S, Cell Signaling Technology), anti-BCL11A (D4E3P, Cell Signaling Technology). After incubation with Dynabeads Protein A or G (Invitrogen, Waltham, MA), samples were washed with hypotonic buffer four times. Proteins were resuspended in 5× Laemmli sample loading buffer, subjected to SDS-PAGE, and transferred to nitrocellulose membranes for immunoblotting.

### Quantitative real-time PCR

Total RNA was extracted using at E.Z.N.A Total RNA kit (Omega Bio-tek, Norcross, GA). 3 μg of RNA was reverse transcribed using Superscript II RT according to the manufacturer's instructions (ThermoFisher), and quantitative PCR was performed using SYBR Green dye (Roche) and the CFX96 Touch Real-Time PCR Detection System (Bio-Rad, Hercules, CA). Primer sequences used in this study are as follows: CDH1 forward: 5′-CCCAATACATCTCCCTTCACAG-3′, CDH1 reverse: 5′-CCACCTCTAAGGCCATCTTTG-3′; CDCA7 forward: 5′- ATGCTTGCAAAACTCATGTCTG-3′, CDCA7 reverse 5′-CTCTCCGTTCAGGGTTTCTC-3′; COL1A2 forward: 5′- AGGACAAGAAACACGTCTGG-3, COL1A2 reverse: 5′- GGTGATGTTCTGAGAGGCATAG-3′; GADD45A forward: 5′-CGTTTTGCTGCGAGAACGAC-3′, GADD45A reverse: 5′- GAACCCATTGATCCATGTAG; DDX18 forward: 5′-GGTTGCTCTGTCATTTGGTTTC-3′, DDX18 reverse: 5′-CTGCTTGCCTTCATTACTGTTG-3′; KISS1R forward: 5′-TTCGTCAACTACATCCAGCAG-3′, KISS1R reverse: 5′-GAACACCGTCACGTACCAG-3′; CDH11 forward: 5′-TGGCAGCAAGTATCCAATGG-3′, CDH11 reverse: 5′-TTTGGTTACGTGGTAGGCAC-3′; CCL7 forward: 5′-GAGAGCTACAGAAGGACCAC-3′, CCL7 reverse: 5′-GTTTTCTTGTCCAGGTGCTTC-3′; EGFR forward: 5′-GGTGGCACCAAAGCTGTATT-3′, EGFR reverse: 5′-GGTGCAGGAGAGGAGAACTG-3′; TRPS1 forward: 5′-GTATCCTGCATCGGGAGAAA-3′, TRPS1 reverse: 5′-AGCTTCTGGTAGAGGCCACA-3′; SLITRK6 forward: 5′-TCCAGTGCTCTCATCCAGAGG-3′, SLITRK6 reverse: 5′-AGTTGGAAAGGTCGTGATGGT-3′; MMP7 forward: 5′-TGAGCTACAGTGGGAACAGG-3′, MMP7 reverse: 5′-TCATCGAAGTGAGCATCTCC-3′; MMP20 forward: 5′-TGAGAGGGGCACTGCTTACT-3′, MMP20 reverse: 5′-GTCTTCTGTGGCTCCCTGAG-3′; HIF-1α forward: 5′-CAGTTACAGTATTCCAGCAGACTCAAA-3′, HIF-1α reverse: 5′-CAGTGGTGGCAGTGGTAGTGG-3′; c-Myc forward: 5′-CAGCGACTCTGAGGAGGAAC-3′, c-Myc reverse: 5′-CCCTCTTGGCAGCAGGATAG-3′; EHF forward: 5′-GCCTTCCATCATGAACACCT-3′, EHF reverse: 5′-GGGTTCTTGTCTGGGTTCAA-3′; VOPP1 forward: 5′-GGCTGTGGTACTTCTGGTTCCTT-3′, VOPP1 reverse: 5′-GTGTAGGACACATTGAAGGCTGG-3′; MX1 forward: 5′-GTTTCCGAAGTGGACATCGCA-3′, MX1 reverse: 5′-CTGCACAGGTTGTTCTCAGC-3′; IFIT1 forward: 5′-TTGATGACGATGAAATGCCTGA-3′, IFIT1 reverse: 5′-CAGGTCACCAGACTCCTCAC-3′; IFIT2 forward: 5′-AAGCACCTCAAAGGGCAAAAC-3′, IFIT2 reverse: 5′-TCGGCCCATGTGATAGTAGAC-3′; IFIT3 forward: 5′- GAACATGCTGACCAAGCAGA-3′, IFIT3 reverse: 5′-CAGTTGTGTCCACCCTTCCT-3′; CXCL11 forward: 5′-GACGCTGTCTTTGCATAGGC-3′, CXCL11 reverse: 5′-GGATTTAGGCATCGTTGTCCTTT-3′; ISG15 forward: 5′-CGCAGATCACCCAGAAGATCG-3′, ISG15 reverse: 5′-TTCGTCGCATTTGTCCACCA-3′; IL6 forward: 5′-CCTGAACCTTCCAAAGATGGC-3′, IL6 reverse: 5′-TTCACCAGGCAAGTCTCCTCA-3′; IRF1 forward: 5′-CTGTGCGAGTGTACCGGATG-3′, IRF1 reverse: 5′-ATCCCCACATGACTTCCTCTT-3′; STAT2 forward: 5′-CCAGCTTTACTCGCACAGC-3′, STAT2 reverse: 5′-AGCCTTGGAATCATCACTCCC-3′; PTPN6 forward: 5′-GGAGAAGTTTGCGACTCTGAC-3′, PTPN6 reverse: 5′-GCGGGTACTTGAGGTGGATG-3′.

### Cell proliferation assay

Cell proliferation was measured by MTT assay. Cells (5 × 10^3^/well) were seeded in 96-well plates in 100 μl DMEM. 3-(4,5-dimethylthiazol-2-yl)-2,5-diphenyltetrazolium (Sigma-Aldrich) solution (20 μl per well, 5 mg/ml in PBS) was added to each well. After incubation at 37°C for 2 h, the supernatant was removed. Formazan was dissolved in DMSO and absorbance was measured using the 540 nm filter of a Victor X5 microplate reader (Perkin Elmer, Waltham, MA).

### Transwell migration assay

Cell migration assays were performed using 8.0 μm pore size Transwell inserts (Costar Corp., Cambridge, MA). Cells were serum-starved overnight (DMEM plus 0.5% FBS), harvested with trypsin/EDTA (Gibco, Carlsbad, CA), and washed twice with serum-free DMEM. 0.6 ml DMEM plus 10% FBS was added in the lower chambers. Cells were re-suspended in medium (DMEM with 0.5% FBS), and 2 × 10^5^ cells in 0.1 ml were added to the upper chambers. After a 12-h incubation at 37°C, the cells on the upper surface of the membrane were removed using cotton tips. The migratory cells attached to the lower surface were fixed in 3.7% formaldehyde and 100% methanol at room temperature for 30 min, and stained with a solution containing 1% crystal violet and 2% ethanol in 100 mM borate buffer (pH 9.0) for 20 min. Five independent fields of view were used to count the number of migratory cells on the lower surface of the membrane under a microscope.

### Drug synergy studies

The synergistic effects of JQ1 and TP-064 were calculated by combination index (CI), based on the enzyme kinetic models of Chou-Talalay (CompuSyn software version 1.0).

### T cell infiltration quantification

Lung tissue from Balb/c mice injected with 4T1.2 cells was collected and washed using ice-cold PBS. The tissue was minced using forceps and scissors into 1–2 mm sections and pressed through a cell strainer (40 μm cell suspension filter). Whole blood was collected and lysed with cold 1 × RBC lysis buffer (155 mM ammonium chloride, 10 mM sodium bicarbonate, 0.1 mM EDTA) for 10–15 min at room temperature. Lung tissue and whole blood were spun down at 250 g for 10 min at 4°C. The resulting pellet was washed twice with PBS. Subsequently, single-cell suspensions were incubated with Ghost Dye Red 780 (Tonbo Biosciences, San Diego, CA) in PBS at 4°C for 30 min. Samples were washed with PBS containing 2% FBS, 2mM EDTA, and 2mM sodium azide, then incubated with antibodies at 4°C for 1 h. Cells were washed with PBS containing 2% FBS, 2 mM EDTA, 2 mM sodium azide, and analyzed using the Attune NxT Flow Cytometer (Thermo Fisher Scientific, Waltham, MA). Flow cytometry beads (eBioscience, San Diego, CA) stained with each antibody were used as single-color controls. A combination of antibodies was used depending on the purpose of each study: CD45 PE (Biolegend, clone 30-F11), CD3 FITC (Biolegend, clone 17A2), CD8a Pe-Cy7 (Biolegend, clone 53-6.7), CD4 APC (Biolegend, GK1.5).

### Cell cycle analysis

Cells were fixed in 70% ethanol at 4°C for 1 h, washed twice with PBS, and incubated with 50 μg/ml propidium iodide (Sigma-Aldrich, St. Louis, MO) and 0.5 μg/ml RNase A in PBS for 4 h. Flow cytometry analysis was performed using an Attune NxT Flow Cytometer at 493/636 excitation/emission (Thermo Fisher Scientific, Waltham, MA). Data analysis was performed using FlowJo (version 7.0).

### Annexin V/PI staining

Cells were washed twice with PBS followed by incubation with annexin V buffer containing annexin V-FITC and propidium iodide (eBioscience, San Diego, CA) for 15 min. Flow cytometry analysis was performed using an Attune NxT Flow Cytometer with excitation at 493/636 (PI) and 490/525 (FITC) excitation/emission (Thermo Fisher Scientific, Waltham, MA). Data analysis was performed using FlowJo (version 7.0).

### Proximity ligation assay (PLA)

MDA-MB-468 cells (1 × 10^5^) seeded on a cover glass in a six-well plate were treated with either JQ1 or TP-064 for 24 h at 37°C in a humidified incubator with 5% CO_2_. Cells were fixed with 4% paraformaldehyde and 100% ice-cold methanol, permeabilized with 0.5% Triton X-100. Several steps including blocking, primary antibody incubation, PLA probe incubation and ligation amplification were performed using a Duolink^®^ In Situ Red Starter Kit (Sigma-Aldrich). Monoclonal BRD4 (E4 × 7E, Cell Signaling) and methyl-BAF155 antibodies were used as primary antibodies or target proteins for PLA. After DAPI staining for 15 min, slides were imaged using the Lionheart FX Automated Microscope (BioTek, Winnoski, VT).

### Immunohistochemistry

Tissues, including tumor, lymph node, and lung, were paraffin-embedded and sectioned by the Experimental Animal Pathology Laboratory (Carbone Cancer Center, University of Wisconsin-Madison; Madison, WI, USA). Tissue sections were deparaffinized and rehydrated using xylenes and ethanol. After antigen retrieval using 10 mM citrate buffer pH 6 and avidin-biotin blocking (Biocare Medical, Pacheco, CA), tissue sections were incubated with primary antibodies (1:500) at 4°C overnight. Secondary biotin-labeled IgG (4 + biotinylated goat anti-rabbit/mouse IgG, Biocare Medical, Pacheco, CA) incubation and Streptavidin–HRP (Biocare Medical, Pacheco, CA) incubation were performed for 15 min at room temperature. Finally, slides were stained with 3,3′-diaminobenzidine (DAB) (Biocare Medical, Pacheco, CA), and counterstained with hematoxylin (Sigma-Aldrich, St. Louis, MO).

### Granzyme B activity assay

Jurkat cells were harvested with RIPA buffer (Thermo Fisher Scientific, Waltham, MA) containing the following protease inhibitors: 1 mM phenylmethylsulfonyl fluoride, 10 μg/ml aprotinin, 1 μM leupeptin, 10 μg/ml pepstatin, 1 mM sodium orthovanadate, 1 mM sodium fluoride. The cells were lysed and sonicated using a Bioruptor (Diagenode, Denville, NJ) for 150 s. After centrifuging at 15,000 rpm for 10 min at 4°C, the supernatant was collected, and protein concentrations were quantified using a Bradford assay. Samples were prepared using a Granzyme B Activity Fluorometric Assay Kit (BioVision, Milpitas, CA) according to the manufacturer's instructions. Fluorescence was measured at Ex/EM = 380/500 mm using a Victor X5 microplate reader (Perkin Elmer, Waltham, MA).

### Live cell imaging

For cell cycle analysis, H2B-RFP-expressing MDA-MB-468 cells were grown on a 4-chambered glass bottom dish (#1.5 glass, Cellvis) in FluoroBrite DMEM media (Thermo Fisher) supplemented 10% FBS (Gibco) and 2 mM GlutaMAX (Gibco). Cells were recorded at 37°C with 5% CO_2_ in a stage-top incubator (TokaiHit). Cells were treated with either DMSO (control), JQ1 (500 nM), or TP-064 (10 μM) for 4 h prior to live cell imaging. Fluorescence images of three conditions were taken at the same time using the Nikon Ti-E microscope, which is equipped with a Nikon intenslight (Nikon), a 40× silicon objective (NA 1.25), and an iXon 888 Life camera (Andor) controlled by Nikon Element software. Time-lapse imaging collected 10 frame 3D stacks at 2 μm steps along the z-axis at 15 min intervals for 24 h. Image analysis was performed using Nikon Element software and Image J software.

For migration assays, H2B-RFP expressing MDA-MB-468 cells were seeded on a glass-bottom slide (μ-slide VI, Ibidi) in FluoroBrite DMEM with an FBS concentration gradient (0-10%) and recorded at 37°C with 5% CO_2_ in a stage top incubator (TokaiHit). Imaging was taken under the same conditions using the same equipment for cell cycle imaging, except with a 40× dry objective (NA/0.8, Nikon). Each treatment condition (control, JQ1, and TP-064 treatment) was imaged on different days to minimize the effects of cell movement (no multi-point function was used). Time-lapse imaging collected 10 frame 3D stacks at 4 min intervals. Tracking analysis of nuclei labeled by H2B-RFP was performed using Imaris 9.5 software (Bitplane).

### Chromatin immunoprecipitation (ChIP) assay

Cells were fixed with 1% formaldehyde for 15 min. Chromatin from the fixed cells was sonicated to obtain 500 bp chromatin fragments. Solubilized chromatin was diluted and incubated incubated at 4°C overnight with the following antibodies: anti-me-BAF155 (10), anti-BAF155 (D7F8S, Cell Signaling Technology), anti-H3K4me1 (Cell Signaling Technology), anti-ARID1A (D2A8U, Cell Signaling Technology), anti-ARID1B (E9J4T, Cell Signaling Technology), anti-ARID2 (D8D8U, Cell Signaling Technology), BRG1 (D1Q7F, Cell Signaling Technology), BCL11A (D4E3P, Cell Signaling Technology), anti-Ac-CBP/p300 (Cell Signaling Technology), anti-p300 (D8Z4E, Cell Signaling Technology), anti-HDAC1 (D5C6U, Cell Signaling Technology), anti-PBRM1 (D3F7O, Cell Signaling Technology), anti-H3K27Ac (Abcam), anti-BRD7 (Abcam), anti-BRD9 (Abcam), anti-BRD4 (Bethyl). After incubation with protein A/G Beads (Invitrogen, Carlsbad, CA) for 2 h, chromatin was washed, eluted and reversed crosslinked. Eluted DNA fragments were purified using a QIAquick PCR Purification Kit (Qiagen, Hilden, Germany) and subjected to quantitative real-time PCR or ChIP-seq. Primer sequences used in study are as follows: CDH1 forward: 5′-ACCCCCTCTCAGTGGCGT-3′, CDH1 reverse: 5′-GGAGCGGGCTGGAGTCTG-3′; CDCA7 forward: 5′-GCAAGTTTTGCTCTTCACGC-3′, CDCA7 reverse: 5′-ATAATCGAGTTCACCGCCCC-3′; COL1A2 forward: 5′-CCTCAACTTCCACAGGGGTC-3′, COL1A2 reverse: 5′-GCTCACTTTATCTCGGGGCA-3′; GADD45A forward: 5′-TGGGTTGCCTGATTGTGGAT-3′, GADD45A reverse: 5′-TAGGGAGTAGCTGGGCTGAC-3′; DDX18 forward: 5′-TGCACCCACAGAGGATAGGA-3′, DDX18 reverse: 5′-CAACAGGAAACGCGTCACAG-3′; NDRG1 forward: 5′-GTAATTGGCTGCTCTTGGCT-3′, NDRG1 reverse: 5′-TATGGGGGATCTAGGCCAGG-3′; TRPS1 forward: 5′-ATCGTCAAGAACACCCTCGG-3′, TRPS1 reverse: 5′-ACAGAAGACGGTTCATGGCT-3′; SLITRK6 forward: 5′-TCCCCATCAGAGCGTTTTAATCT-3′, SLITRK6 reverse: 5′-AGTTGAGCAGTCCCAAGGTG-3′; MMP7 forward: 5′-CCTCAGGGGAGGTCCAAGTG-3′, MMP7 reverse: 5′-TCCACAACCCACAAATGGAGT-3′; MMP20 forward: 5′-TGCCATTAGAACTGTGGCTTG-3′, MMP20 reverse: 5′-TGCCATTAGAACTGTGGCTTG-3′; HIF-1α forward: 5′-GGAATGCGTGGTCTGGGTAA-3′, HIF-1α reverse: 5′-TACAACATTCCCGCTCTGCC-3′; c-Myc forward: 5′-GGAATGCGTGGTCTGGGTAA-3′, c-Myc reverse: 5′-TACAACATTCCCGCTCTGCC-3′; EHF forward: 5′-GGGCAGATGCCTTTCTTTGC-3′, EHF reverse: 5′-TGCTCCGATAACAACGCAGT-3′; IL6 forward: 5′-CCTGAACCTTCCAAAGATGGC-3′, IL6 reverse: 5′-TTCACCAGGCAAGTCTCCTCA-3′; IRF1 forward: 5′-CTGTGCGAGTGTACCGGATG-3′, IRF1 reverse: 5′-ATCCCCACATGACTTCCTCTT-3′; STAT2 forward: 5′-CCAGCTTTACTCGCACAGC-3′, STAT2 reverse: 5′-AGCCTTGGAATCATCACTCCC-3′; PTPN6 forward: 5′-GGAGAAGTTTGCGACTCTGAC-3′, PTPN6 reverse: 5′-GCGGGTACTTGAGGTGGATG-3′; IFIT3 forward: 5′-CCCTACTCTCCCACCCCTTT-3′, IFIT3 reverse: 5′-CTGTGTCTCTGCTGTTCCGA-3′; MX1 forward: 5′-AATCATAGCAAGGGCGCTGA-3′, MX1 reverse: 5′-AGGGGGATGTTTCTGATGCG-3′; IFIT2 forward: 5′-CTGAGGAGAGAGCGATCCGA-3′, IFIT2 reverse: 5′-GTTTGAAACCAAGGCCGACG-3′; CXCL11 forward: 5′-ACAGAAGAATAGGCGGCGAG-3′, CXCL11 reverse: 5′-CCTACATCGAGGAGGTGGGA-3′; IFI27 forward: 5′-GCTTTGTCTCAGGCACGAAC-3′, IFI27 reverse: 5′-CCTTGTCCGGTCATCAGAGC-3′; ISG15 forward: 5′-AGGATCTGGAATGCGCGATA-3′, ISG15 reverse: 5′-GGAAAAGCAAAAGTGGCGGG-3′; RSAD2 forward: 5′-GGAAACGAAAGCGAAGCGTT-3′, RSAD2 reverse: 5′-CGTTTATCGCGCACATCTCG-3′.

### Chromatin immunoprecipitation coupled to deep sequencing (ChIP-seq) analysis

Purified DNA (or Input DNA) was quantified using Qubit 4 (Thermo Fisher Scientific, Waltham, MA). The ChIP-seq library was prepared using the Ovation Ultralow System V2 (NuGEN Technologies, Redwood City, CA) according to the manufacturer's instructions. Libraries were sequenced either with an Illumina HiSeq 4000 using 50 bp reads (Illumina, San Diego, CA) at the NUSeq Core at Northwestern University Feinberg School of Medicine or using an Illumina HiSeq 2500 (Illumina) using 50 bp reads at the University of Wisconsin-Madison Biotechnology Center.

### RNA sequencing (RNA-seq)

Total RNA was isolated using the E.Z.N.A. Total RNA kit (Omega Bio-tek) from MDA-MB-468 cells, as well as HCI-002, HCI-009 and 4T1.2 tumor tissues. The RNA-seq library was prepared in triplicate using a TruSeq RNA Library Prep Kit v2 (Illumina, San Diego, CA) according to the manufacturer's instructions. The libraries were sequenced either with an Illumina HiSeq 4000 (Illumina) using 50 bp reads at the NUSeq Core at Northwestern University Feinberg School of Medicine or using an Illumina HiSeq 2500 (Illumina) using 50 bp reads at the University of Wisconsin-Madison Biotechnology Center.

### Paramagnetic particle preparation

50 μg paramagnetic particles (PMPs) (Sera-mag SpeedBeads Streptavidin-Blocked, GE) were used for each sample. The PMPs were washed three times and resuspended in PBS containing 0.1% Tween-20 (Thermo Fisher). Biotinylated antibodies of epithelial cell adhesion molecule (EpCAM, R&D Systems) and trophoblast cell-surface antigen 2 (Trop-2, R&D Systems) were then added to the washed PMPs and mixed for 30 min at room temperature. After three washes, PMPs were resuspended in PBS with 10% FBS (Thermo Fisher).

### Automated CTC isolation and staining

To bind CTCs in the CD45^–^ cell fraction, the antibody conjugated PMPs were added to the CD45^–^ cell fraction and incubated for 30 minutes under constant rotation at 4°C, before being loaded onto the automated Gilson ExtractMax robot. The magnetic beads and cell mixture were then moved magnetically though a series of staining and wash steps. The cells were stained extracellularly for 20 min with Hoechst and antibodies against CD45 (Biolegend, H130), CD34 (Biolegend, 581) and CD66b (Biolegend, GF10F5) in a PBS and 10% FBS staining buffer. Next, cells were pulled into a Foxp3 Fixation and Permeabilization Solution (F/P, Invitrogen), diluted per manufacturer protocols, and incubated for 20 min. Next, cells were moved into an intracellular staining solution comprised of Foxp3 Permeabilization Wash Buffer (P/W, Invitrogen), pCK (Biolegend, C-11), and a 1:500 unconjugated me-BAF155 antibody for 20 min. Subsequently, cells were incubated in the secondary staining solution which consists of 1:200 pre-conjugated Goat anti-Rabbit IgM antibody (Bethyl Laboratories, A120-210D4) in P/W for 20 min. After three washes with P/W and one wash with PBS, samples were transferred to a glass bottom chamber slide (Electron Microscopy Sciences), and settled for 30 min at 4°C prior to imaging.

### CTC Image acquisition

Images were acquired of the entire imaging chamber by acquiring a grid of 15 × 15 small image tiles, then all tiles together to create one large image per sample. Individual image tiles were acquired using a 20× objective of the Nikon Eclipse Ti-E fluorescent microscope (Nikon) in multiple wavelengths including bright field (BF), 350 nm (Hoechst), 560 nm (me-BAF155), 647 (Exclusion) and 790 (cytokeratin, pCK). To ensure uniform focus for each individual tile, the step-by-step focus setting was used with 2 μm intervals across a 10 μm range, with a 2 μm offset to shift the focal plane from that of the magnetic beads to that of the larger cells. Re-focusing each individual image ensures uniform focus is maintained across the entire imaging chamber to overcome any slight stage tilt in the *Z*-plane.

### Image analysis

Images were analyzed with NIS-Elements AR version 4.51 (Nikon). Average background fluorescence was subtracted from each channel using the rolling ball algorithm with a size 50 rolling ball. Cells were then cataloged by using binary layer objects to define threshold algorithm and by using the intensity of the Hoechst stain to define the edges of each cell. To exclude items that were too small, too large, or too blurry, restrictions were applied for parameters of cell size, circularity, and intensity variation, respectively. CTCs were defined as cells that were positive for Hoechst, and pCK, and negative for exclusion channel markers. Once the CTCs were identified, the CTC binary layer was eroded to leave a solely nuclear CTC binary layer. The mean fluorescence intensity (MFI) of the me-BAF155 staining of each CTC nuclear region was then exported into Microsoft Excel for graphical analysis in GraphPad Prism.

### Bioinformatics analysis

ChIP-seq reads were aligned to human genome (hg19 assembly, excluding Chromosome Y) by bwa (version 0.7.15). ChIP-seq peaks were called by MACS (version 2.1.0) with a q-value cutoff of 0.05. Peaks were filtered by removing those overlapping with ENCODE’s exclusion list regions (https://www.encodeproject.org/files/ENCFF001TDO/) and those not on Chromosomes 1–22 or X. ChIP-seq signals were normalized by read depth and by the length of the corresponding genomic region. Screenshots of ChIP-seq signals at gene loci were from UCSC Genome Browser using the smoothing option of ‘smoothing Window 10’. Super-enhancers were computed by the ROSE package (http://younglab.wi.mit.edu/super_enhancer_code.html) based on BRD4 ChIP-seq peaks overlapping with H3K27Ac peaks from the same treatment, as well as ChIP-seq read alignments of BRD4 and its corresponding control. Annotation of transcripts’ exons and introns was from ROSE. Promoters were defined as 5 kb upstream of a transcript's transcription start site, and not overlapping with any exons or introns. The remaining genomic regions were defined as distal locations. A ChIP-seq peak was assigned to a gene if it overlaps with this gene's promoter, exon, or intron. KEGG pathways enriched of genes with both BRD4 and meBAF155 ChIP-seq peaks were inferred by the kegga function from the R package limma (version 3.44.3). Peak binding near the TSS region at ±2k bp and motif analysis shared between me-BAF155 and BRD4 binding data was performed using Homer software (version 4.10.4).

RNA-seq reads were aligned by STAR (version 2.5.2b) either to the human genome (hg19 assembly excluding Chromosome Y) with transcript annotations from ROSE or to the mouse genome (mm10 assembly excluding Chromosome Y and the four unplaced chromosomes associated with Y) with basic GENCODE gene annotations (version M22). Gene expression levels were quantified by RSEM (version 1.3.0), and differential expression was analyzed by DESeq2 (version 1.22.1). A differentially expressed gene was required to have at least two-fold changes and an adjusted *P*-value <0.05. Gene set enrichment analysis was performed by the cameraPR function from the limma package with the 50 Hallmark gene sets from the Molecular Signatures Database (version 6.1). Gene Set Enrichment Analysis (GSEA) from 125 SE-signature genes and IFN α pathway genes was performed using GSEA software (version 4.0.3). A heatmap of DEGs of IFN α pathway genes was created by GItools (version 2.3.1). A scatter plot comparing MDA-MB-468 and HCI-002 samples under JQ1 or CARM1i treatment was created using DEGs of IFN α, γ and α/γ pathway genes.

### Statistical methods and software

Mann-Whitney U and two tailed t tests were used to compare two independent group. The Log-rank test was used for the survival curve analyses based on Kaplan–Meier methods. All analyses were performed using GraphPad Prism software 5.0. *P* < 0.05 was considered of significance. The sample size (*n*) represents biological replicates in cell culture experiments and number of mice in animal studies.

## RESULTS

### Me-BAF155 genomic binding sites largely overlap with those of BRD4 at super-enhancers

**Figure 1.**
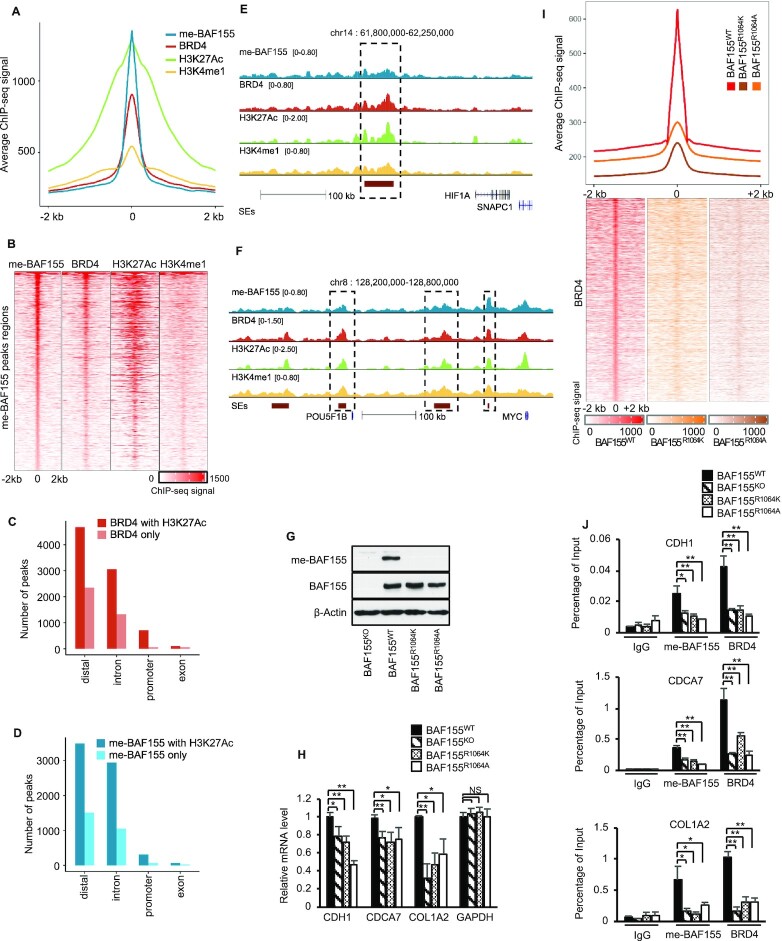
Overlap of me-BAF155 and BRD4 genomic binding sites at super-enhancers and BRD4 SE association depends on BAF155 methylation at R1064. (**A**) Average ChIP-seq signals of me-BAF155, BRD4, H3K27Ac and H3K4me1 around me-BAF155 peaks in MDA-MB-468 cells treated with vehicle. Shown are 2kb flanking regions of me-BAF155 peak summits. (**B**) ChIP-seq signals of me-BAF155, BRD4, H3K27Ac and H3K4me1 around me-BAF155 peak regions in MDA-MB-468 cells treated with vehicle. Peak regions of me-BAF155 are the same as in (A). (**C**, **D**) Number of BRD4 and meBAF155 peaks stratified by their genomic locations and overlapping with H3K27Ac peaks in MDA-MB-468 cells treated with vehicle. (**E**) Me-BAF155, BRD4, H3K27Ac and H3K4me1 ChIP-seq signals at *HIF1A* in MDA-MB-468 cells. (**F**) Me-BAF155, BRD4, H3K27Ac and H3K4me1 ChIP-seq signals at *MYC* in MDA-MB-468 cells. (**G**) Generation of BAF155 KO MDA-MB-231 cells using CRISPR/Cas9 and restoration of BAF155^WT^ and two methyl-defective mutants BAF155^R1064K^ and BAF155^R1064A^. (**H**) Real-time qPCR analyses of CDH1, CDCA7 and COL1A2 levels in cell lines in panel G. Data are mean ± s.d. **P< 0.01; *P< 0.05; NS: not significant. (**I**) BRD4 ChIP-seq signals in MDA-MB-231 cells expressing BAF155^WT^, BAF155^R1064K^ and BAF155^R1064A^. Signals were computed in the 2kb flanking regions of BRD4 peak summits in cells expressing BAF155^WT^. (**J**) Binding of me-BAF155 and BRD4 to CDH1, CDCA7, and COL1A2 genes measured by ChIP-qPCR in cell lines in Panel G. Data are mean ± s.d. **P< 0.01; *P< 0.05.

We have previously shown that me-BAF155 promotes cancer metastasis in TNBC models (10). To interrogate the mechanism of me-BAF155-dependent cancer metastasis, we mapped the genomic binding sites of me-BAF155 in MDA-MB-468, a TNBC cell line. Of the total 9,475 me-BAF155 binding sites identified by ChIP-seq, 4,393 me-BAF155 peaks overlapped with the 4,314 peaks of BRD4 (out of a total of 12,364 peaks). The overlapping peak regions were also enriched with H3K27Ac and H3K4me1, two chromatin signatures of super-enhancers (SEs) (Figure 1A and B). Accordingly, BRD4/H3K27Ac, or me-BAF155/H3K27Ac co-occupied peaks, were largely localized in the distal regions (Figure 1C and D). There was significant enrichment of me-BAF155, BRD4, H3K27Ac and H3K4me1 peaks within the SE regions of *HIF1A* and *MYC*, two representative oncogenes in TNBC (Figure 1E and F). Transcription factor (TF) motif analyses revealed that AP2, TEAD and bZIP were among the top TFs in these genomic regions (Supplementary Figure S1A). To investigate if BRD4 association with SEs is dependent on BAF155 methylation, we knocked out (KO) BAF155 using CRISPR/Cas9 and re-expressed wild-type (WT) BAF155 or two methyl-defective mutants, BAF155^R1064K^ and BAF155^R1064A^, in MDA-MB-231 cells (Figure 1G and Supplementary Figure S1B). Q-RT-PCR analyses of previously identified metastasis-related genes (10) confirmed the down-regulation of metastasis inducers and up-regulation of metastasis repressors in BAF155 KO or BAF155 mutant-expressing cells (Figure 1H and Supplementary Figure S1C). Next, we mapped BRD4 genomic occupancies in these functionally validated cell lines. The results showed that BRD4 occupancies were dramatically diminished in BAF155^R1064K/R1064A^-expressing MDA-MB-231 cells, as compared to parental cells (Figure 1I). Several previously identified CARM1-regulated metastasis-related genes were validated by ChIP-q-PCR. The results showed that me-BAF155 and BRD4 occupancies on these genes were significantly decreased in BAF155 KO or BAF155 methyl-defective mutants, but not in MDA-MB-231 expressing BAF155^WT^ (Figure 1J and Supplementary Figure S1D), demonstrating that me-BAF155 regulates BRD4 association with SEs. Collectively, our results reveal that me-BAF155 not only co-occupies genome binding sites with BRD4 and H3K27Ac, but also directly affects BRD4 association with SEs.

### CARM1 inhibitor (CARM1i) treatment dissociates BRD4 and me-BAF155 from SEs, reduces SE numbers, and inhibits expression of SE-regulated oncogenes

**Figure 2.**
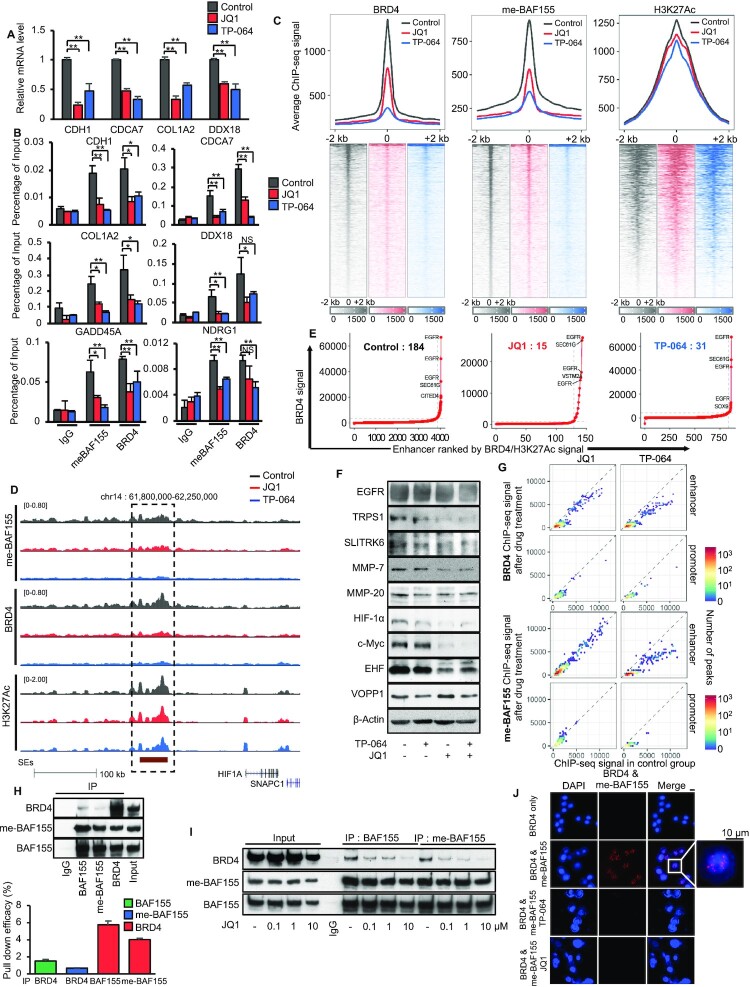
CARM1 inhibitors eradicate BRD4 and me-BAF155 binding sites at SEs and decrease the expression of oncogenes addicted to SEs. (**A**) mRNA levels of me-BAF155 target genes (CDH1, CDCA7, COL1A2 and DDX18) after vehicle, JQ1, or TP-064 treatment in MDA-MB-468 cells measured by q-RT-PCR. **P< 0.01. (**B**) ChIP-qPCR analyses of me-BAF155 and BRD4 association with indicated genes after vehicle, JQ1, or TP-064 treatment in MDA-MB-468 cells. **P< 0.01; *P< 0.05; NS: not significant. (**C**) BRD4, me-BAF155 and H3K27Ac ChIP-seq signals in MDA-MB-468 cells after treatment with vehicle (*black*), JQ1 (*red*) or TP-064 (*blue*) in 2kb flanking regions of me-BAF155 peak summits from vehicle-treated cells. (**D**) ChIP-seq signals of me-BAF155, BRD4 and H3K27Ac in vehicle, JQ1 or TP-064 treatment conditions near *HIF1A*. (**E**) Super-enhancer signal-to-rank plots for MDA-MB-468 cells treated by vehicle, JQ1 or TP-064. The numbers of SEs were decreased from 184 to 15 and 31 by JQ1 and TP-064 treatment, respectively. (**F**) Western blotting of representative SE-regulated oncoproteins in vehicle, JQ1, TP-064, or JQ1 plus TP-064 treatment conditions in MDA-MB-468 cells. (**G**) BRD4 (top) and me-BAF155 (bottom) ChIP-seq signals before and after drug treatment. Signals were calculated in BRD4 (top) and me-BAF155 (bottom) peaks from MDA-MB-468 cells treated by vehicle. (**H**) Co-immunoprecipitation assays was performed using IgG, BRD4, me-BAF155, and BAF155 antibodies from nuclear lysates of MDA-MB-468 cells. Western blotting was performed using BRD4, me-BAF155, and BAF155 antibodies (top). The pull-down efficiency of each antibody was normalized by the band intensities of immunoprecipitates over the input using densitometry of the western blot bands (*n* = 3). (**I**) Decreased BRD4-association with BAF155 or me-BAF155 by increasing the amount of JQ1 in co-IP assays using MDA-MB-468 cells. Nuclear lysates were precipitated using BAF155 or me-BAF155 antibodies and co-precipitated BRD4 was detected by western blotting. (**J**) Proximity ligation assays showing the interaction between BRD4 and me-BAF155 was sensitive to JQ1 and TP-064 treatment in MDA-MB-468 cells.

Because Bromodomain and extra-terminal domain (BET) proteins regulate an array of cancer-associated genes and pathways, pharmacological inhibition of BET proteins using inhibitors (BETi) has emerged as an anti-inflammatory and anti-cancer epigenetic therapeutic modality (20). To further interrogate the functional relationship between me-BAF155 and BRD4, we treated TNBC cells with BETi JQ1 or CARM1i TP-064 (14), followed by measuring me-BAF155 and BRD4 chromatin occupancies, respectively. As a control, we validated that mRNA levels of me-BAF155-regulated metastasis genes were attenuated by either JQ1 or TP-064 treatment in MDA-MB-468 cells (Figure 2A), which is in accordance with the reduced me-BAF155 and BRD4 binding on these genes (Figure 2B). Similar effects of these drugs were observed in MDA-MB-231 (Supplementary Figure S2A and B). We then measured me-BAF155, BRD4, and H3K27Ac genomic occupancies after treatment with DMSO, JQ1 or TP-064 in MDA-MB-468 cells. Figure 2C shows that both me-BAF155 and BRD4 peaks were dramatically reduced, whereas H3K27Ac peak enrichment on SEs was slightly decreased by TP-064 (*P* = 0.007) and not affected by JQ1 (*P* = 0.07), respectively. For example, both me-BAF155 and BRD4 peaks were dramatically decreased by JQ1 or TP-064 treatment on *HIF-1A* and *MYC* (Figure 2D and Supplementary Figure S2C). SE-regulated oncogenes were defined as the closet genes to an SE that have overlapping BRD4 and H3K27Ac peaks. Under these stringent criteria, we identified 184 SE-regulated oncogenes, and JQ1 or TP-064 treatment decreased SE numbers to 15 and 31, respectively, in MDA-MB-468 cells (Figure 2E and Supplementary Figure S2D). To exclude the possibility that the decrease in SEs is TP-064-specific, we employed another CARM1-specific inhibitor, EZM2302, which is orally bioavailable, and has been shown to elicit *in vivo* anti-cancer effects in multiple myeloma (12). First, we confirmed that EZM2302 inhibited arginine methylation on two CARM1-specific substrates, BAF155 and PABP1 (21), in a dose-dependent manner (Supplementary Figure S2E), and that EZM2302 treatment dissociated BRD4 from chromatin at the SE cluster of *HIF1A* (Supplementary Figure S2F). Next, we performed BRD4 ChIP-seq in the presence of EZM2302. The results showed that EZM2302 dramatically decreased BRD4 genomic occupancy (Supplementary Figure S2G). Strikingly, the BRD4 dissociation sites induced by EZM2302 or TP-064 treatment were almost identical (Supplementary Figure S2H). We selected several SE-regulated oncogenes, such as *SLITRK6*, *TRPS1*, *EHF* and *MMP-7* from Supplementary Figure S2D for validation. Western blotting results showed that protein levels of these genes were decreased by either JQ1 or TP-064 treatment (Figure 2F). As expected, both BRD4 and me-BAF155 genomic sites were mainly clustered on enhancers, but less in promoters (Figure 2G). Further analysis of JQ1 or TP-064-induced changes in me-BAF155 and BRD4 genomic sites revealed that TP-064 abrogated both BRD4 and me-BAF155 genomic occupancies in promoter and enhancer regions, whereas JQ1 was only effective at decreasing BRD4 occupancy, but had less of an effect on me-BAF155 binding (Figure 2G and Supplementary Figure S2I). These results demonstrate that inhibiting BAF155 methylation has profound effects on BRD4 recruitment to SEs, whereas inhibiting BRD4 with JQ1 does not affect me-BAF155 genomic binding.

To investigate whether regulation of BRD4 genomic occupancy at SEs by me-BAF155 requires their interaction, we performed co-immunoprecipitation assays using me-BAF155, total BAF155, or BRD4-specific antibodies. The results showed that BRD4 can pull-down BAF155 and me-BAF155, and *vice versa* (Figure 2H, upper). However, only a small fraction of BRD4 and BAF155 appeared to form a complex, as normalization of immunoprecipitated proteins to input in western blots performed in triplicate revealed that <6% of BRD4 was pulled down by antibodies against BAF155 or me-BAF155, and <2% of BAF155 and ∼1% of me-BAF155 were pulled down by BRD4 antibody (Figure 2H, bottom). Moreover, the interaction between me-BAF155 and BRD4 was attenuated by treatment with JQ1 (Figure 2I) or TP-064 (Supplementary Figure S2J). To further define the interaction between me-BAF155 and BRD4, we performed a proximity ligation assay (PLA) in the presence of JQ1 or TP-064 and found that me-BAF155 co-localized with BRD4 in nuclear puncta (Figure 2J), resembling phase-separated condensates enriched in co-activators (17). Treatment with either JQ1 or TP-064 decreased the interaction between BRD4 and me-BAF155 (Figure 2J). The BRD4-containing condensates could be abolished by treatment with 1,6-hexanediol (1,6-HD), an aliphatic alcohol that can immobilize liquid droplets (17). Supplementary Figure S2K shows that nuclear BRD4 immunofluorescence staining was abolished by treatment with either TP-064 or 1,6-HD, in MDA-MB-468 cells. Together, our data demonstrate that BRD4 association with SEs is largely dependent on BAF155 methylation, possibly through physical interaction between me-BAF155 and BRD4. Therefore, CARM1 inhibition dissociates me-BAF155 and BRD4 from SEs, inhibiting SE-regulated oncogene expression.

### Despite low cytotoxicity, CARM1 inhibition blocked cell migration of TNBC cell lines and elicited synergistic effects with JQ1

**Figure 3.**
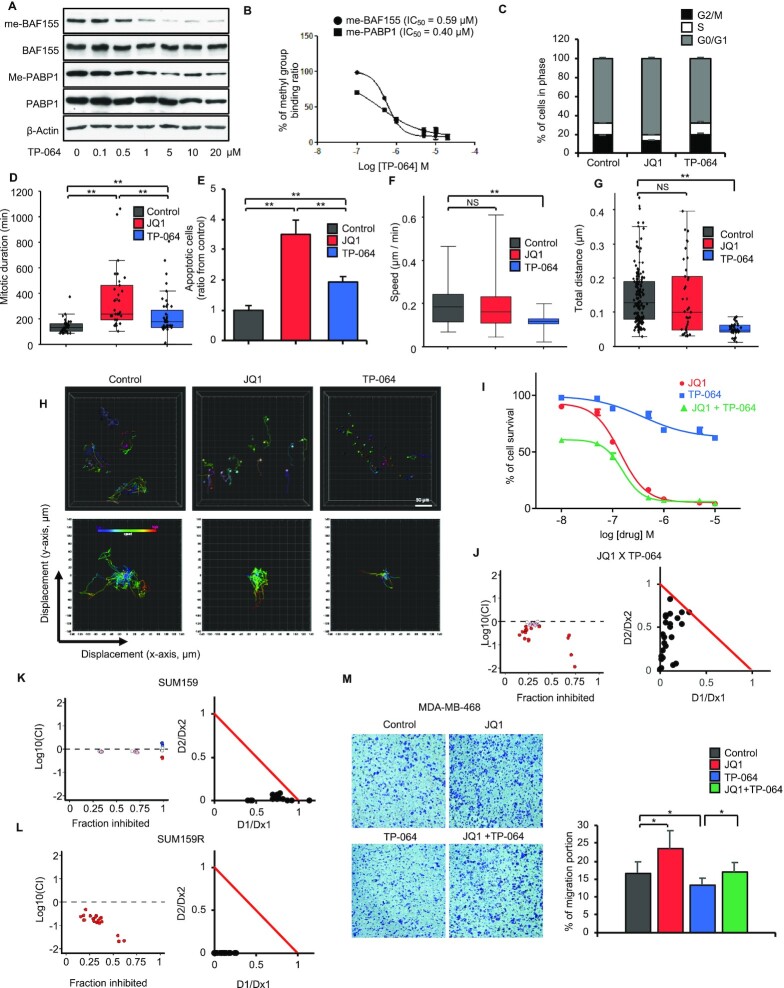
TP-064 exhibits low cytotoxic effects, inhibits cell migration, and synergizes with JQ1 in triple-negative breast cancer cells. (**A**) Dose-dependent inhibition of CARM1-mediated methylation of BAF155 and PABP1 by TP-064. Total and methyl-specific BAF155 and PABP1 antibodies were used for immunoblotting of MDA-MB-468 total lysate. (**B**) Calculation of IC_50_ for TP-064 inhibited BAF155 and PABP1 methylation based on the band intensities in panel A. (**C**) MDA-MB-468 cell cycle analyses after treatment with vehicle, JQ1 or TP-064. (**D**) JQ1 but not TP-064 treatment increases mitotic duration. Average duration of mitosis was measured by time-lapse imaging after JQ1 or TP-064 treatment in H2B-RFP labeled MDA-MB-468 cells. Data are mean ± s.d. **P< 0.01, *n* = 33–42 cells. (**E**) Normalized apoptotic index by JQ1 or TP-064 in MDA-MB-468 cells. The number of apoptotic cells after treatment with JQ1 or TP-064 were normalized with those in vehicle treated condition. Data are mean ± s.d. **P< 0.01. (**F**) Average speed (μm/min) during cell migrations after treatment with JQ1 or TP-064 in H2B-RFP labelled MDA-MB-468 cells. Data are mean ± s.d. **P< 0.01; NS: not significant. (**G**) Average of distance (μm) of single cell migration over a 24-hour period after JQ1 or TP-064 treatment in MDA-MB-468 cells. Data are mean ± s.d. **P< 0.01; NS: not significant. (**H**) Representative images of single cell migration over time in control, JQ1, and TP-064 treated H2B-RFP expressing MDA-MB-468 cells (top) and plots of 2D displacements (bottom). (**I**) MDA-MB-468 cell viability in response to ascending doses of JQ1, TP-064 or JQ1 and TP-064 combination. (**J–L**) Synergistic growth inhibitory effect of JQ1 and TP-064 in MDA-MB-468 (J) and SUM159 (K) cells and CARM1 inhibits cell proliferation in SUM159R cells (L). Synergy was calculated by combination index (CI) based on enzyme kinetic models of Chou-Talalay for JQ1 and TP-064 combination (Supplementary Figure S3C and F). CI plot was represented by fraction inhibited (x-axis) and CI value substituted by log10 (y-axis). For normalized isobologram plot, CI scores are plotted by D1/Dx1 and D2/Dx2, where Dx1 (JQ1) and Dx2 (TP-064) are the doses of each single drug, causing fractional inhibition effect x, respectively. The red line indicates a boundary of additive effect. (**M**) Transwell cell migration assays after treating MDA-MB-468 cells with vehicle, JQ1, TP-064 or their combination. Migrated cells were stained with 1% crystal violet (left) and the percent of migrated cells under treatment of vehicle, JQ1, TP-064 or TP-064 plus JQ1 were plotted (right). Data are mean ± s.d. *P< 0.05.

The inhibitory effect of CARM1i on BRD4 and me-BAF155 genomic association and oncogene expression prompted us to assess the functional effects of CARM1i in proliferation and migration, in comparison with JQ1, which is known to elicit strong cytotoxic effects in TNBC cells (22). First, we determined the IC_50_ of TP-064 to inhibit BAF155 and PABP1 in MDA-MB-468 cells. Western blot results showed dose- and time-dependent inhibition of BAF155 and PABP1 methylation (Figure 3A and Supplementary Figure S3A). Similar to TP-064’s IC_50_ to inhibit methylation of BAF155 in HEK293 cells (0.34 μM) (14), the IC_50_ for inhibiting methylation of BAF155 and PABP1 was determined to be 0.59 and 0.40 μM, respectively (Figure 3A and B). Thus, we used 10 μM TP-064 for subsequent cell-based assays because BAF155 methylation is expected to be completely inhibited by TP-064 at this concentration. Next, we examined the effects of TP-064 and JQ1 on the cell cycle. TP-064 did not influence the cell cycle, whereas JQ1 treatment resulted in G0/G1 arrest in MDA-MB-468 cells (Figure 3C). To compare the effects of JQ1 and TP-064 on mitosis, we employed H2B-GFP-tagged MDA-MB-468 cells and tracked the duration in mitosis and migration of individual cells using a high-resolution microscope (Supplementary Movies 1–3 for DMSO, JQ1 and TP-064). The results showed that mitotic duration times were similar between TP-064 and vehicle treated groups, whereas JQ1 increased mitotic duration time at single-cell resolution (Figure 3D). JQ1 treatment also increased apoptotic cell death and abnormal cell division, whereas TP-064 treatment had little effect (Figure 3E and Supplementary Figure S3B). Finally, H2B-GFP labeling of MDA-MB-468 cells allowed us to assess the effects of JQ1 and TP-064 on cell migration, which is an indicator of cancer metastasis (23), at the single-cell level using microscopy. The results showed that TP-064 significantly decreased migratory speed and moving distance as compared to vehicle and JQ1 treatment (Figure 3F–H). The ability of TP-064 to inhibit cell migration is consistent with our previous finding that BAF155 methylation drives cell migration in TNBC cells (10).

BETi has been extensively investigated for combination treatment with other therapeutic agents, including CDK inhibitors, HDAC inhibitors, and PD-1 inhibitors (20). Thus, we evaluated if JQ1 and CARM1i elicit synergistic effects on cell proliferation and migration. We measured the cytotoxic effects of TP-064 and JQ1 alone, and in combination, in a dose-dependent manner (Figure 3I and Supplementary Figure S3C). The results showed that TP-064 has few cytotoxic effects, even at 10 μM, whereas JQ1 has strong cytotoxic effects in MDA-MB-468 cells. When combined, cell survival was significantly decreased as compared with JQ1 or TP-064 alone (Figure 3I). To examine if JQ1 and TP-064 elicit synergistic effects on cell survival, we calculated the combination effect value based on the Chou-Talalay method (24). The results showed that JQ1 and TP-064 acted synergistically to inhibit cell proliferation in MDA-MB-468 cells (Figure 3J), as well as in SUM159 (Figure 3K), another JQ1-sensitive TNBC cell line (25). Interestingly, SUM159R, a JQ1 resistant cell line, remained responsive to TP-064 (Figure 3L). The results suggest that although TP-064 alone displayed low cytotoxic effects, it synergizes with JQ1 to inhibit cell growth in multiple TNBC cell lines. The growth inhibitory effects of TP-064 in SUM159R cells implies that TP-064 might be effective in JQ1-resistant cells.

To compare the effects of TP-064 and JQ1 on cell migration, we performed transwell migration assays. Surprisingly, TP-064 inhibited, while JQ1 promoted, migration of MDA-MB-468 cells (Figure 3M). However, JQ1’s effects on cell migration appear to be cell-line specific, as JQ1 inhibited migration of SUM159, but not of JQ1-resistant SUM159R cells (Supplementary Figure S3D). The combination of JQ1 and TP-064 inhibited migration synergistically in SUM159 cells, as shown by the calculated CI value (Supplementary Figure S3E). Treatment with TP-064, but not JQ1, inhibited migration of SUM159R cells (Supplementary Figure S3F). These results suggest that although both JQ1 and CARM1i inhibited common targets (i.e., oncogenes regulated by SEs), these two inhibitors affect non-overlapping targets and pathways. Hence, the combination of BETi and CARM1i elicited synergistic anti-growth and anti-migratory effects, and CARM1i remains effective in JQ1-resistant cells.

### BET and CARM1 inhibitors abrogated growth and metastasis of patient-derived xenografts

**Figure 4.**
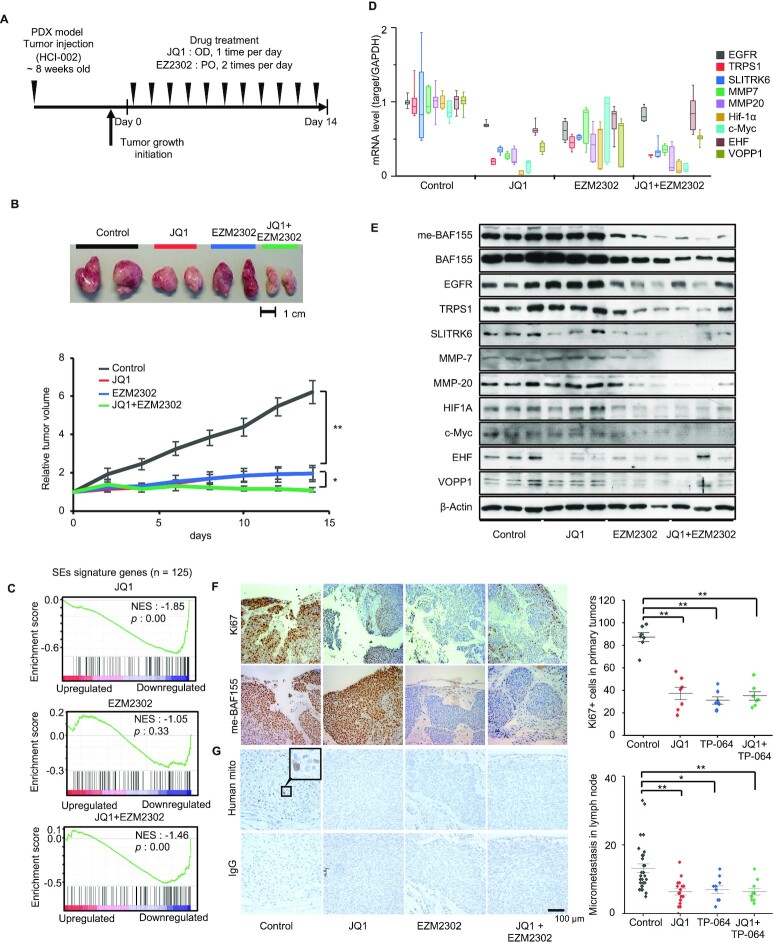
Inhibition of HCI-002 PDX tumor growth and metastasis by JQ1 and EZM2302. (**A**) A schematic of the workflow treating HCI-002 xenografts with vehicle, JQ1, EZM2302, or both. (**B**) Representative tumors in vehicle, JQ1, EZM2302 or both treatment groups (*Top*). Growth curves (*bottom*) show the tumor volumes normalized by the pre-drug treated tumor volumes. **P< 0.01; *P< 0.05. (**C**) Gene set enrichment analysis (GSEA) of RNA-seq data (*n* = 3) from tumors treated with JQ1, TP-064 or JQ1 and TP-064 in combination on 125 SEs signature genes. (**D**) Real-time qPCR analyses of SE-regulated genes after treatment with vehicle, JQ1, EZM2302 or both in HCI-002 PDX. (**E**) Western blotting of SE-regulated oncoproteins after treatment with indicated drugs in HCI-002 PDX. (**F**) Ki67 (*upper left*) and me-BAF155 (*lower left*) IHC staining in vehicle, JQ1, EZM2302 or both treated HCI-002 tumors. Ki67-positive cells under each treatment condition were plotted (*right*). Data are mean ± s.d. **P< 0.01. (**G**) Detection of lymph node micrometastasis by IHC using human specific mitochondria antibody (*upper left*) with HCI-002 tumors. IgG serves as a negative control (*lower left*). Micrometastatic cancer cells in lymph nodes were quantified under each treatment conditions (*right*). Data are mean ± s.d. **P< 0.01; *P< 0.05.

We next determined the *in vivo* effects of BETi and CARM1i in HCI-002, a patient-derived xenograft (PDX) model representing TNBC (26). Because EZM2302 is orally bioavailable and has been shown to elicit anti-cancer effects in other cancer models (12,13), we treated HCI-002 PDX tumors with JQ1, EZM2302, alone, or in combination, as shown in the scheme of Figure 4A. Treatment with JQ1, EZM2302, alone, or in combination, significantly reduced HCI-002 tumor growth (Figure 4B). The body weights of mice were largely unaffected by drug treatment. JQ1-treated mice experienced some weight loss within the first week of treatment, but then recovered as the study progressed (Supplementary Figure S4A). Since both inhibitors targeted SEs and decreased expression of SE-regulated oncogenes, we collected tumors from each treatment group for RNA-seq analyses. Gene Set Enrichment Analysis (GSEA) revealed that either JQ1, TP-064, or their combination, down-regulated expression of SE-signature genes in HCI-002 (Figure 4C). A similar result was observed in MDA-MB-468 cells (Supplementary Figure S4B). To determine if inhibition of SE-signature gene transcripts by JQ1 or TP-064 is coupled with decreased protein levels, we examined the mRNA and protein levels of several SE-regulated oncogenes in HCI-002 tumors by Q-RT-PCR and western blot, respectively. Indeed, SE-regulated genes were inhibited by either drug alone or their combination (Figure 4D and E). As a positive control, me-BAF155 levels were decreased by EZM2302 or EZM2302 and JQ1, but not by JQ1 alone. The intensity of Ki67 staining was significantly decreased by either JQ1 or EZM2302, indicating the anti-proliferative effects of these drugs in HCI-002 (Figure 4F). HCI-002 has been reported to form micrometastases in lymph nodes (26). Lymph node tissues were harvested from mice treated with JQ1, EZM2302, or both, and stained with a human-specific mitochondria antibody. Both drugs were found to inhibit micrometastasis as compared vehicle treatment (Figure 4G). To exclude the possibility that the effects are HCI-002-specific, we repeated the experiment in HCI-009, another TNBC PDX (Supplementary Figure S4C). Like HCI-002, HCI-009 tumor growth was inhibited by either JQ1 or EZM2302 without detectable body weight changes (Supplementary Figure S4D and E). Additionally, genes within a SE gene signature and selected SE-regulated genes were downregulated after drug treatment (Supplementary Figure S4F and G), coinciding with the decrease in onco-protein levels (Supplementary Figure S4H). The HCI-009 model has been reported to metastasize to lung (26). Thus, we detected metastatic cells by staining human antigens in lung tissues. The results showed that either JQ1 or TP-064 decreased lung metastasis (Supplementary Figure S4I and J). Collectively, our results demonstrate that inhibition of BET or CARM1 significantly blocks tumor growth and metastasis in PDX models, possibly through inhibiting expression of SE-regulated oncogenes because this mechanism is shared between BRD4 and me-BAF155.

### Differential effects of BET and CARM1 inhibitors in the 4T1 syngeneic mouse model

**Figure 5.**
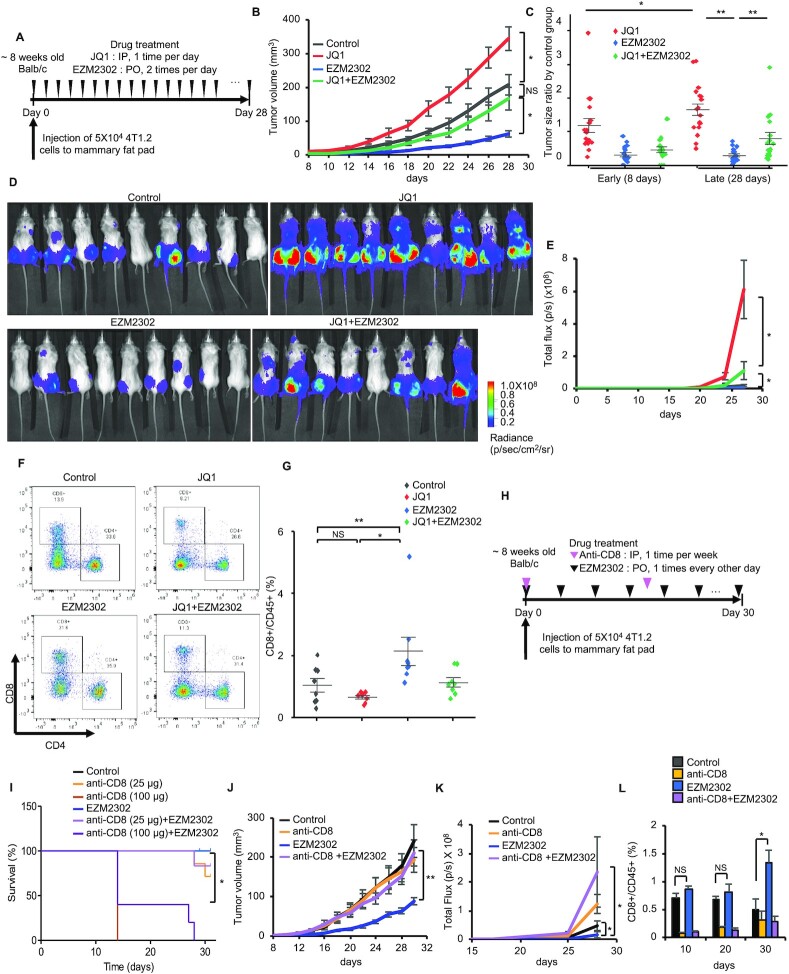
EZM2302 inhibits, but JQ1 promotes, 4T1.2 lung metastasis and their differential effects on the regulation of CD8^+^ T cells. (**A**) A schematic of the workflow using vehicle, JQ1, EZM2302, or both for treatment of 4T1.2 orthotopic tumors. (**B**) 4T1.2 tumor growth curve under indicated treatment conditions (vehicle, JQ1, EZM2302, or both). Data are mean ± s.e.m. *n* = 9 (vehicle, JQ1, EZM2302 groups) and *n* = 8 (JQ1 + EZM2302 group). *P< 0.05; NS: not significant. (**C**) Drug-induced tumor volume changes normalized to the vehicle-treated group at 8 days or 28 days. Data are mean ± s.d. **P< 0.01; *P< 0.05. (**D**) Bioluminescence imaging of 4T1.2-luciferase tumors in mammary fat pads and lung after treatment with vehicle, JQ1, EZM2302, or both for 28 days. (**E**) Luciferase signal intensities of 4T1.2 tumor cells metastasize to lung in the indicated treatment groups. Data are mean ± s.d. *P< 0.05. (**F**) Flow cytometry analyses of CD4 and CD8 expressing cells from lungs of 4T1.2 syngeneic mouse models under indicated treatments. (**G**) Quantification of CD8^+^ T cells from CD45^+^ immune cells from lungs of 4T1.2 syngeneic model under indicated treatments. **P< 0.01; *P< 0.05; NS: not significant. (**H**) A schematic of antibody and drug treatment investigating the role of CD8^+^ T cells in 4T1.2 tumor growth. 4T1.2 tumors were treated with vehicle, EZM2302, anti-CD8 antibody or both, starting from the day of 4T1.2-luciferase injection to mammary fat pad. (**I**) Kaplan–Meier survival curves of 4T1.2 tumor bearing mice in indicated treatment groups. Two doses (25 and 100 μg) of anti-CD8 antibody were used. *P< 0.05. (**J**) Growth curve of primary 4T1.2 tumors under indicated treatment conditions. **P< 0.01. (**K**) Lung metastases detected by bioluminescence imaging of four treatment groups. *P< 0.05. (**L**) Quantification of CD8^+^/CD45^+^ cells from peripheral blood of control, anti-CD8, EZM2302 or anti-CD8 plus EZM2302 treatment groups after 10, 20 and 30 days. **P< 0.01; *P< 0.05; NS: not significant.

Although both JQ1 and EZM2302 inhibited growth and metastasis in a PDX model, PDX models lack an intact immune system. To examine the *in vivo* effects of JQ1 and EZM2302 in an immune-competent breast tumor model, we orthotopically implanted 4T1.2, a mouse cell line that can spontaneously metastasize (27,28), into syngeneic Balb/c mice. 5 × 10^4^ 4T1.2-luciferase cells were injected to the inguinal fat pads of 8-week-old female Balb/c mice. Mice were randomized to treatment with either JQ1, EZM2302, or a combination of both agents following the treatment regimen depicted in Figure 5A. Tumor volume was measured using calipers, and metastasis to the lungs was measured by bioluminescence imaging (BLI) (Figure 5A). Compared to vehicle treatment, EZM2302 significantly decreased tumor growth (Figure 5B and C). Surprisingly, JQ1 dramatically increased tumor growth. However, co-treatment with EZM2302 offset JQ1’s tumor promoting effects (Figure 5B and C). Moreover, JQ1 promoted, while EZM2302 inhibited, lung metastasis of 4T1.2 tumor cells, as detected by BLI (Figure 5D and E). To examine if pre-treatment with JQ1 or EZM2302 has chemopreventive effects on 4T1.2 tumor growth, we pre-treated mice with either JQ1, EZM2302, or a combination of both drugs for three days prior to implanting 4T1.2 cells to mimic tumor recurrence after surgery (Supplementary Figure S5A). The results showed that EZM2302 still significantly inhibited tumor growth and lung metastasis (Supplementary Figure S5B–D). Interestingly, although JQ1 did not promote local tumor growth as compared to the vehicle-treated group, JQ1 still promoted lung metastasis of 4T1.2 tumors (Supplementary Figure S5B–D). The addition of EZM2302 to JQ1 offset JQ1’s metastasis-promoting effects to a similar level as the control group (Supplementary Figure S5C and D). This result agrees with the migration-promoting effect of JQ1 in MDA-MB-468 (Figure 3M).

The incongruous results seen in immunodeficient PDX models and the 4T1.2 syngeneic model in response to JQ1, where JQ1 inhibited metastasis in the PDX models and promoted metastasis in the 4T1.2 model, imply that immune cells might play an important role in cancer metastasis (29,30). We surmise that BETi and CARM1i might have different effects on immune cells. To test this hypothesis, we quantified the number of tumor infiltrating lymphocytes (TILs) present in the lung tissue of 4T1.2 tumor-bearing mice by flow cytometry. Indeed, treatment with EZM2302 increased CD8^+^ T cells in the lungs (Figure 5F and G). To investigate whether targeted blockade of CD8 antagonizes or abrogates the therapeutic effects of EZM2302, we treated 4T1.2 tumors with EZM2302 alone, or in combination with an anti-CD8 mAb, followed by measuring survival, tumor growth, metastasis, and CD8^+^ T cell infiltration. Two doses of anti-CD8 mAb (25 or 100 μg) (30) were employed to ensure the viability of mice at least in the low-dose anti-CD8 group. Figure 5H shows the treatment scheme. Indeed, rapid tumor growth was observed in the anti-CD8 treatment group. When a higher dose of anti-CD8 mAb was administered, the mice died within two weeks (Figure 5I), whereas EZM2302 prolonged survival. Thus, we monitored primary tumor growth and metastasis in the low-dose anti-CD8 mAb, EZM2302, and the combination treatment groups (Figure 5J and K). The results showed that EZM2302 significantly inhibited tumor growth. However, this effect was abrogated by co-treatment with anti-CD8 mAb (Figure 5J). Higher 4T1.2 metastasis measured by BLI was also observed in the anti-CD8, or anti-CD8 and EZM2302 co-treatment groups, as compared with the vehicle and EZM2302 treatment groups (Figure 5K). We further quantified tumor infiltrating CD8^+^/CD45^+^ T cells under treatment conditions over time (10, 20 and 30 days). The results showed that anti-CD8 mAb decreased CD8^+^ T cells, as expected (Figure 5L). EZM2302 treatment increased blood (Supplementary Figure S5E) and local tumor infiltrating CD8^+^ T cells (Figure 5L) as compared with vehicle treatment after 30 days, although the effects did not reach statistical significance at the earlier time points. The anti-CD8 mAb abrogated the CD8^+^ T cell-activating effects of EZM2302 (Figure 5L). Moreover, neither treatment caused body weight changes (Supplementary Figure S5F). To interrogate the effects of EZM2302 on T cell proliferation and activity, we employed an *in vitro* co-culture system consisting of TNBC cells and Jurkat T cells. Jurkat cells were co-cultured with or without MDA-MB-468 cells, followed by treatment with JQ1, TP-064 alone, or a combination of both drugs. Treatment with JQ1 decreased CD8^+^ T cell numbers as compared with the vehicle and TP-064-treated groups (Supplementary Figure S5G). Surprisingly, granzyme B activity, which is an indicator of T cell cytotoxic activity (31), was dramatically increased by TP-064, but inhibited by JQ1, in Jurkat cells when co-cultured with MDA-MB-468 (Supplementary Figure S5H). These data strongly suggest that CARM1i enhanced, whereas JQ1 inhibited, the cytotoxic effects of CD8^+^ T cells, which explains the differential effects of these epigenetic drugs in the immunocompetent 4T1.2 model.

### CARM1 inhibition alleviates repression of Interferon (IFN) α/γ pathway genes by decreasing me-BAF155 and increasing association of BCL11A, PBAF subunits and H3K27Ac levels at IFN α/γ pathway genes

**Figure 6.**
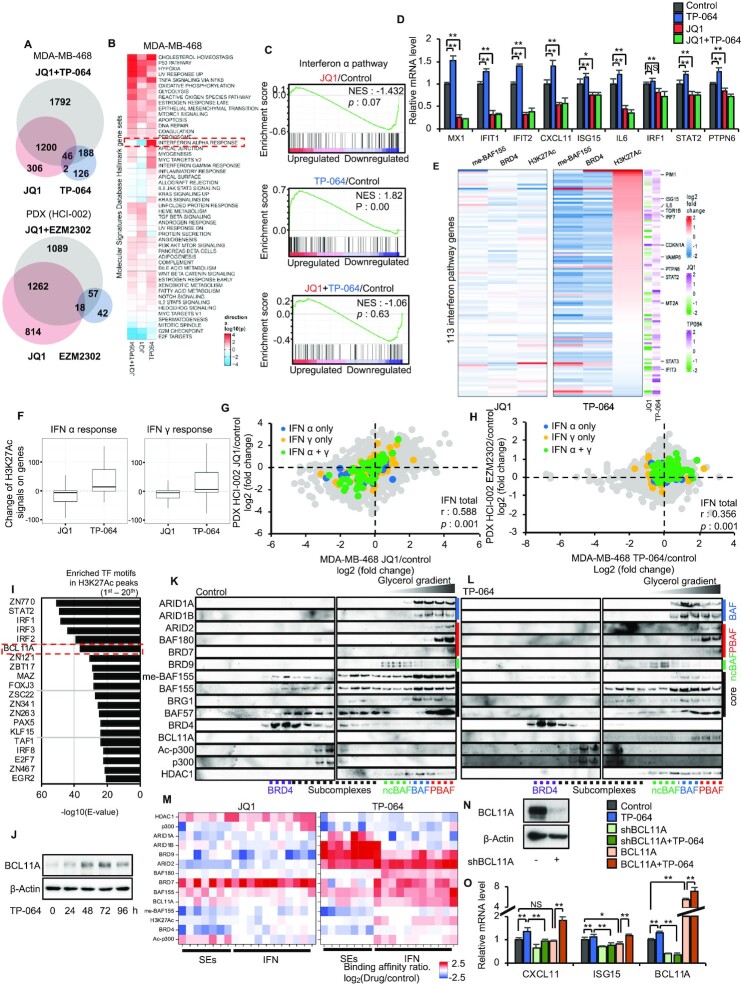
CARM1 inhibitors activate IFN α/γ pathway genes via inducing the formation of BCL11A/PBAF complex and elevating H3K27Ac levels. (**A**) Venn diagram showing the overlap of DEGs induced by JQ1, CARM1 inhibitor or JQ1 + CARM1 inhibitor in MDA-MB-468 cells (*top*) or HCI-002 (*bottom*). (**B**) Heatmap showing Hallmark gene sets up- (red) or down-regulated (blue) by JQ1, TP-064 or JQ1 + TP-064 treatment in MDA-MB-468 cells. (**C**)GSEA of IFN α pathway signature genes induced by JQ1, TP-064 or JQ1 and TP-064 in combination in MDA-MB-468 cells. (**D**) Q-RT-PCR analyses of indicated IFNα pathway genes after treatment with JQ1, TP-064 or JQ1 and TP-064 in combination in MDA-MB-468 cells (*n* = 3). Data are mean ± s.d. **P< 0.01; *P< 0.05; NS: not significant. (**E**) Increased (red) and decreased (blue) me-BAF155, BRD4, and H3K27Ac ChIP-seq signals after TP-064 or JQ1 treatment in MDA-MB-468 cells on IFN α and γ genes that had increased expressions (magenta) after TP-064 treatment. Many of the IFN genes have expression level decreased (blue) after JQ1 treatment. (**F**) Changes of H3K27Ac ChIP-seq signals on genes from the Hallmark IFNα (*left*) and IFNγ (right) response gene sets induced by JQ1 or TP064. (**G, H**) Scatter plot showing the correlation of mRNA level changes between MDA-MB-468 (x-axis) and HCI-002 tumors (y-axis) by JQ1 (G) or TP-064/EZM2302 (H). IFN α pathway only genes are depicted in blue dots, IFN γ pathway only genes are depicted in yellow dots, and IFN α/γ shared pathway genes are depicted in green dots. (**I**) Enriched transcription factor motifs at H3K27Ac peaks of the promoters and gene bodies of IFN pathway genes. (**J**) Immunoblotting of BCL11A protein levels after treatment of MDA-MB-468 cells with TP-064 for indicated time. (**K, L**) Western blotting of indicated proteins from nuclear extracts of MDA-MB-468 cells by glycerol density gradient (10–45%) after vehicle (K) or TP-064 (L) treatment. (**M**) Summary of ChIP-qPCR data of indicated proteins binding to SEs or IFN pathway genes after JQ1 (*left*) or TP-064 (*right*) treatment in MDA-MB-468 cells. Red depicts high level and blue depicts low level binding of indicated proteins (y-axis), respectively. (**N**) Western blotting of BCL11A in parental or BCL11A knockdown MDA-MB-468 cells. (**O**) Q-RT-PCR analyses of IFNα pathway genes after treatment with DMSO or TP-064 in parental, BCL11A KD and overexpressing MDA-MB-468 cells. **P< 0.01; *P< 0.05; NS: not significant.

The increased tumor infiltration and cytotoxicity of CD8^+^ T cells seen under EZM2302 treatment prompted us to examine immune responses in breast cancer cell lines, PDXs, and 4T1.2 tumors. We performed RNA-seq using MDA-MB-468 cells, HCI-002 tumors, and 4T1.2 tumors to identify differentially expressed genes (DEG) induced by JQ1, CARM1i or both. Compared to the other groups, CARM1i (TP-064 in MDA-MB-468 or EZM2302 *in vivo*) treatment induced lower numbers of DEG (Figure 6A and Supplementary Figure S6A), and this DEG (362 in MDA-MB-468, 117 in HCI-002, 64 in 4T1.2) fell into either activated or repressed gene categories (Supplementary Figure S6B and C). This contrasts with JQ1, which induced larger numbers of DEGs across all three models (1554 in MDA-MB-468, 2094 in HCI-002, 878 in 4T1.2). Although there are DEGs specific to the JQ1 and CARM1i co-treatment condition, the DEGs in the JQ1 and CARM1i co-treatment group largely overlap with those of JQ1 alone (Supplementary Figure S6A and B), suggesting that JQ1 is the dominant regulator of gene expression when JQ1 and CARM1i are combined. Strikingly, GSEA revealed that JQ1 inhibited interferon (IFN) responsive pathways, whereas these pathways were strongly activated by CARM1i across three different models (Figure 6B, C and Supplementary Figure S6D and E). IFN α/γ stimulated genes (ISGs) were activated by CARM1i, as shown in the heatmap, (Supplementary Figure S6F) and validated by qRT-PCR (Figure 6D). On the contrary, ISGs were inhibited by treatment with JQ1 alone, or its combination with CARM1i (Figure 6D and Supplementary Figure S6F). To explore the mechanism for the differential regulation of ISGs by CARM1i and JQ1, we integrated RNA-seq results into me-BAF155, BRD4, and H3K27Ac ChIP-seq data of ISGs in response to TP-064 and JQ1 treatment in MDA-MB-468 cells. Interestingly, a subset of ISGs had elevated H3K27Ac, and me-BAF155 and BRD4 genome occupancies were decreased by TP-064 treatment (Figure 6E and F). JQ1, on the contrary, inhibited expression of many ISGs, accompanied by slightly decreased or unchanged levels of H3K27Ac (Figure 6E and F). JQ1-mediated inhibition and CARM1i-mediated activation of ISGs were conserved *in vitro* and *in vivo*, as shown by the strong correlation between the MDA-MB-468 and HCI-002 data for JQ1 (Figure 6G) and CARM1i (Figure 6H) treatment. TP-064 activated ISGs in MDA-MB-468 cells, and EZM2302 activated ISGs in HCI-002 (Figure 6H). These data suggest that CARM1i activates IFN α/γ pathway genes across multiple TNBC models, possibly through elevation of H3K27Ac levels at these gene loci.

To interrogate the mechanism(s) involved in the CARM1i-induced increase in H3K27Ac levels, we analyzed TF binding motifs at the H3K27Ac ChIP-seq peaks of ISGs. The top TFs associated with H3K27Ac peaks included ZN770, STAT2, IRFs and BCL11A (Figure 6I). Western blotting results showed that the levels of STAT1, 2, and IRF1 were unchanged in response to either JQ1 or TP-064 treatment in MDA-MB-468 cells (Supplementary Figure S6G). Interestingly, BCL11A protein (Supplementary Figure S6G) and mRNA levels (Supplementary Figure S6H) were up-regulated by TP-064 and down-regulated by JQ1. Moreover, TP-064 induced a time-dependent increase of BCL11A protein (Figure 6J). BCL11A has been found amplified/overexpressed in TNBC, and is reported to play roles in differentiation in stem and progenitor cells (32). Moreover, BCL11A was shown as an integral subunit of mammalian SWI/SNF complexes (33), namely BRG/BRM-associated factor (BAF), polybromo-associated BAF (PBAF), and non-canonical BAF (ncBAF) complexes (34). Therefore, we posit that BCL11A activates ISGs in response to CARM1i. Because BRG1-containing SWI/SNF has been shown to activate IFN-α-inducible genes (35) and IFN-γ-activated genes (36), we examined the association between BCL11A and SWI/SNF complexes by immunoprecipitation using anti-BCL11A and anti-BAF155 antibodies after treatment with vehicle, JQ1, TP-064, and their combination. Interestingly, TP-064 treatment led to pull-down of more PBAF complex subunits (e.g. BAF180, BRD7 and ARID2) by either BCL11A or BAF155 antibody as compared to control (Supplementary Figure S6I). In contrast, JQ1 treatment had no effects on PBAF subunit co-immunoprecipitation. Neither drug caused detectable changes in BCL11A or BAF155 co-immunoprecipitating with BAF (e.g. ARID1A and ARID1B) or ncBAF (e.g. BRD9) specific subunits (Supplementary Figure S6I). These results imply that TP-064 induces increased levels of BCL11A protein that primarily associate with the PBAF complex.

To further confirm if BCL11A is preferentially associated with the PBAF complex under TP-064 treatment, different forms of the SWI/SNF complex were separated from nuclear lysates of MDA-MB-468 using a glycerol density gradient after DMSO or TP-064 treatment. Under vehicle treatment, me-BAF155 had broad sedimentation profiles with two peaks: one overlapped with that of BRD4 at the low molecular weight range (corresponding to the initial BAF core), the other largely co-eluted with BAF and PBAF, as has been reported (37). TP-064 treatment reduced me-BAF155 to an undetectable level. As a result, total BAF155 was not detected in BRD4-containing fractions using an anti-BAF155 antibody, but instead, was found in fractions containing BAF and PBAF subunits (Figure 6K), suggesting that BAF155’s association with BRD4 could be attributed to methylated BAF155, and this association does not require the entire SWI/SNF complex. BCL11A levels were drastically increased by TP-064 treatment (Figure 6J and Supplementary Figure S6G). Consequently, BCL11A was undetectable in the vehicle-treated glycerol gradient fractions, but was detectable in PBAF fractions when MDA-MB-468 cells were treated with TP-064 (Figure 6K and L). To interrogate the role of BCL11A in regulating ISGs, we performed ChIP-q-PCR assays using the indicated antibodies at specific chromatin regions (SEs and interferon-responsive genes), after DMSO or TP-064 treatment. Increased association of BCL11A was found at ISGs, but to a lesser extent at SE sites, along with BAF155 and PBAF subunits after TP-064 treatment (Figure 6M). Furthermore, TP-064 treatment triggered dissociation of HDAC1, and increased levels of Ac-p300 and H3K27Ac on ISGs, coinciding with TP-064-induced activation of IFN responsive genes.

To determine if BCL11A is required for TP-064-induced ISG activation, we knocked down BCL11A in MDA-MB-468 cells (Figure 6N) and measured mRNA levels of representative ISGs by q-RT-PCR. The results showed that knocking down BCL11A abrogated TP-064 activation of ISGs (Figure 6O). To further interrogate if increased BCL11A alone is sufficient to restore ISG expression, we transduced BCL11A in MDA-MB-468 cells with and without TP-064 treatment (38). The results showed that exogenous expression of BCL11A alone is insufficient to increase expression of ISGs, but it restores, if not further potentiates, TP-064’s ability to active ISGs (Figure 6O). The data suggest that BCL11A expression is necessary but not sufficient for ISG activation, and higher levels of ISG induction require not only BCL11A expression, but also CARM1 inhibition.

IFN pathway activation by CARM1i could be attributed to loss of arginine methylation on various substrates including BAF155. We reasoned that if BAF155 methylation is required for suppressing BCL11A and IFN pathway genes, activation of ISGs should be observed in BAF155 KO and BAF155 methyl-defective mutant expressing MDA-MB-231 cells. Indeed, western blotting results showed that BCL11A protein (Supplementary Figure S6J) and mRNA levels (Supplementary Figure S6K) were elevated in BAF155 KO, BAF155^R1064K^,and BAF155^R1064A^, as compared to BAF155^WT^-expressing MDA-MB-231 cells. Concomitantly, mRNA levels of ISGs increased in BAF155 KO, BAF155^R1064K^, and BAF155^R1064A^ cells (Supplementary Figure S6K). These data strongly support that BCL11A and ISG activation is repressed by me-BAF155 in TNBC cells. In response to CARM1 inhibition or ablation of BAF155 methylation, BCL11A is activated and likely associates with PBAF to activate ISGs by inducing histone acetylation (i.e., increased recruitment of Ac-P300 and dissociation of HDAC1). The activation of IFN α/γ pathway genes through this mechanism leads to enhanced immune responses and increased anti-growth and anti-metastasis effects.

### Detection of me-BAF155 in circulating tumor cells (CTCs) of metastatic breast cancer patients

**Figure 7.**
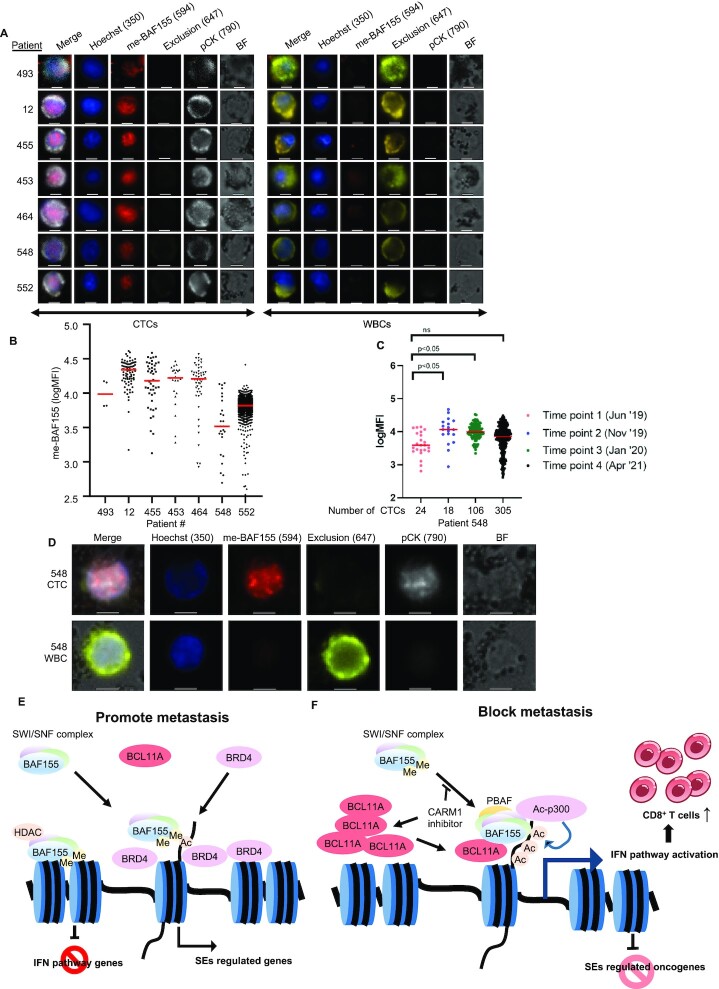
Immunostaining of me-BAF155 in CTCs of metastatic breast cancer patients. (**A**) Images of me-BAF155 immunostaining in the nucleus of CTCs but not in WBCs from seven breast cancer patients. Representative images of CTCs (*left*) and WBCs (*right*) are shown for each patient (ID listed to the left). Each row of tiles includes an image crop of the intensity distribution of each of the stains included in the panel (Hoechst, me-BAF155, Exclusion (CD45/CD34/CD66b), pCK and Bright Field (BF)) for one single cell, including a merge of all stains. Images were taken at 20x magnification; scale bars represent 10 μm. (**B**) The average me-BAF155 nuclear signal intensities in breast cancer patient CTCs. Each dot represents the average me-BAF155 nuclear staining intensities of each individual CTC from one of the seven patients. The red bars indicate the average me-BAF155 nuclear signals of all CTCs from an individual patient sample. (**C**) The tracing of me-BAF155 nuclear signal intensities measurement in CTCs from breast cancer patient 548. The number of CTCs are depicted in x-axis. (**D**) Representative immunofluorescence images of CTCs stained with Hoechst, me-BAF155, Exclusion (CD45/CD34/CD66b), pCK and merged image of all staining signals for patient 548 in April 2021. Scale bars represent 10 μm. (**E, F**) Models depicting dual functions of me-BAF155 dependent metastasis in TNBC cells and anti-metastasis effects by CARM1 inhibition. Me-BAF155 and BRD4 interact at SEs to activate oncogenes and me-BAF155 containing SWI/SNF complex interacts with HDAC1 to suppress ISGs (E). CARM1 inhibitor treatment leads to increased BCL11A and un-methylated BAF155, which assemble with other SWI/SNF subunits to form PBAF to activate ISGs. Un-methylated BAF155 triggers dissociation of BRD4 from SEs, resulting in inhibition of expression of SE-addicted oncogenes (F).

Our results demonstrate that inhibition of me-BAF155 leads to decreased expression of SE-regulated oncogenes, activation of ISGs in tumor cells, as well as enhanced CD8^+^ T cell infiltration and cytotoxicity. These changes due to me-BAF155 inhibition result in ablating metastasis in TNBC models. Because circulating tumor cell (CTC) number is a predictor of metastasis, we posit that me-BAF155 may be detectable in CTCs and used as a prognostic biomarker. Supplementary Figure S7A illustrates our CTC isolation, staining, and image acquisition workflow known as VERSA (versatile exclusion-based rare sample analysis) (39). Targeted cells, such as CTCs or white blood cells (WBCs), were isolated from blood using magnet beads attached to EpCAM antibodies, immunostained for proteins of interest, and imaged with a fluorescence microscope (Supplementary Figure S7B and C). EpCAM, an epithelial cell surface marker, was used to capture CTCs from CD45-excluded peripheral blood (39). Prior to performing me-BAF155 immunofluorescence (IF) in CTCs, we optimized a protocol for IF using a me-BAF155 antibody and BAF155 KO MDA-MB-231 cells as a negative control (Supplementary Figure S7D) (10). Strong nuclear me-BAF155 staining was observed in MDA-MB-231 and MDA-MB-468, but not in BAF155 KO MDA-MB-231 cells (Supplementary Figure S7D and E). Blood was collected from seven metastatic breast cancer patients (3 TNBC, 4 ER+, clinical details listed in Supplementary Table S1), and EpCAM+ cells were captured, and stained with Hoechst (nuclei), or with antibodies targeting cytokeratin, me-BAF155, and exclusion markers (CD45, CD34 and CD66b) (Figure 7A). The results showed that for all patients, me-BAF155 could be detected in CTCs that were positively stained with cytokeratin. However, the intensities of me-BAF155 staining were significantly lower in WBCs. Quantification of me-BAF155 immunostaining in CTCs is shown in Figure 7B. We monitored the me-BAF155 levels of patient BC-548, who had stable TNBC over a three-year period. IF results revealed that, although the number of CTCs significantly increased over time, me-BAF155 levels remained stable over the three-year period (Figure 7C). Figure 7D shows the representative images of CTC staining using different markers for patient BC-548 in April of 2021.

## DISCUSSION

In this study, we uncovered two novel mechanisms depicting how methylation of BAF155 drives breast cancer metastasis. Me-BAF155 directly interacts and cooperates with BRD4 to regulate expression of oncogenes addicted to SEs in TNBC cells. Moreover, me-BAF155 suppresses ISG expression in tumors, and blocks T cell infiltration to metastatic sites.

SEs are widely co-opted by cancer cells to overexpress proteins encoded by oncogenes (e.g. c-Myc) (18). Our finding of me-BAF155’s genomic occupancies at SEs agrees with the established role of SWI/SNF in targeting SEs in other systems (40,41). Intriguingly, me-BAF155 specifically interacted with BRD4 in TNBC cells, based on our three different biochemical assays (Figures 2H, J, 6K and L). Most importantly, inhibition of BAF155 methylation using CARM1i (both EZM2302 and TP-064) eradicated nearly all BRD4 binding at SEs (Figure 2C). Recently, coactivators such as BRD4 with intrinsically disordered regions (IDRs) were shown to form phase-separated condensates at SEs to consolidate transcription apparatus for gene regulation (17). Interestingly, BAF155 methylation site R1064 resides in the proline-enriched C-terminus of BAF155, which presumably forms an IDR (17). The colocalization of me-BAF155 with BRD4 in nuclear puncta (Figure 2J) and the depletion of BRD4 in nuclear puncta by CARM1i in TNBC cells (Supplementary Figure S2K) imply that me-BAF155 likely facilitates the formation of lipid-like condensates and recruits BRD4 for activation of SE-addicted oncogenes.

The predominant mechanistic basis for employing BETi for cancer treatment is the downregulation of MYC. Consistent with our finding (10), CARM1i strongly inhibited expression of a broad array of SE-regulated oncogenes including MYC (Figure 2D and F), resembling the effects of JQ1. The clinical application of BETi has faced major obstacles (42), including low efficacy, strong adverse effects (43), and drug resistance frequently found in solid tumors (44). These drawbacks, at present, preclude their approval by the FDA. Although CARM1i and BETi both regulate SEs, there are many fundamental differences between these two inhibitors. First, in contrast to JQ1, CARM1i elicited negligible cytotoxicity, and did not induce cell cycle arrest and apoptosis. Second, TP-064 inhibited cell migration in all TNBC cell lines tested in this study, while JQ1 increased migration in MDA-MB-468 cells. Third, although both JQ1 and EZM2302 inhibited tumor growth and metastasis in two PDX models, JQ1 promoted, while EZM2302 inhibited, metastasis in 4T1.2 syngeneic mouse models. The low cytotoxic effects of CARM1i in breast cancer cell lines have been reported previously by us and others (14,15). Given their low cytotoxicity and strong anti-metastasis effects, pharmacological inhibitors of CARM1 should be further exploited as anti-cancer drugs for the treatment of broad types of solid tumors in which CARM1 is overexpressed. Our RNA-seq analyses revealed that fewer DEGs were affected by CARM1i as compared to JQ1 in MDA-MB-468, as well as in the HCI-002 PDX and 4T1.2 *in vivo* models. These results further support that CARM1i's anti-cancer and anti-metastatic effects *in vivo* are likely attributed to inhibiting BAF155 methylation by CARM1, as opposed to an off-target effect.

Our data that JQ1 promotes metastasis in 4T1.2 syngenetic mouse models agrees with a recent study in prostate cancer *in vivo* models (45). The metastasis-promoting effect of JQ1 in different systems calls for precaution when clinically developing BETi as cancer therapeutics. The opposing effects of JQ1 on inhibiting metastasis in immunodeficient mouse models and promoting metastasis in an immunocompetent mouse model imply that the immune system plays an important role in metastasis. We found that JQ1 inhibited, whereas EZM2302 increased, tumor infiltrating CD8^+^ T cells in the 4T1.2 model. DEGs of cell lines and tumors treated with JQ1 or CARM1i revealed a clear difference in the regulation of IFN α/γ pathway genes by these compounds, where JQ1 repressed, but CARM1 inhibitor activated, expression of ISGs. BETi has been shown to exhibit anti-inflammatory effects in various systems (46). For example, BETi suppressed cytokine-induced inflammation in monocytes (47). The inhibition of the ISGs in tumor cells by JQ1 likely accounts for the metastasis-promoting effects of JQ1 in the 4T1.2 model. This is in sharp contrast to EZM2302, which activates ISGs, increases CD8^+^ T cell infiltration to tumors, and enhances the cytotoxic effect of CD8^+^ T cells, although the number of CD4^+^ T cells and macrophages remains unchanged (Figure 5F and data not shown). While this manuscript was in preparation, Kumar *et al.* identified CARM1 as a negative regulator of T cell immunity from a CRISPR/cas9 screen (48). They showed that CARM1 inactivation by genetic knockout or by pharmacological inhibition in T cells enhanced anti-tumor T cell function. Our results agree with Kumar *et al.* that CARM1 inhibition enhanced type 1 interferon response in tumors, enhancing T cell-mediated tumor immunity and potentiating anti-metastasis effects. Importantly, we revealed that BAF155 methylation is required for ISG activation (discussed below). Because CARM1i also exhibited strong anti-growth and anti-metastasis effects in immunocompromised PDX models, CARM1 inhibition may regulate additional immune cell functions.

BCL11A is a kruppel-like transcription factor that plays essential roles in lymphoid development and fetal-to-adult hemoglobin switching (49). Recently, BCL11A was found amplified in nearly 40% of basal-like breast tumors (32). However, how BCL11A in TNBC cells is regulated remains unknown. We found that BCL11A expression is induced by TP-064 and is required for CARM1i-induced activation of ISGs. The ISG activation by BCL11A is distinct from its most characterized role as a transcriptional repressor via direct interaction with RBBP4 (50), a component of repressive chromatin complexes that include histone deacetylase SIN3A. BCL11A has been identified as an auxiliary subunit of mammalian SWI/SNF in a proteomics study (33). Our data support that CARM1i treatment leads to HDAC1 dissociation from ISGs, increased BCL11A levels, and PBAF and Ac-p300 association to activate IFN α/γ pathway genes.

The PBAF complex regulates ISGs, although positive or negative regulation appears to be cell-type and context-dependent. ARID2 is required for activation of a subset of IFNα-induced genes in hepatocellular carcinoma (HCC) (51). Moreover, inactivation of PBRM1/BAF180 reduced IFNγ-STAT1 activity in renal carcinoma (52). These studies suggest that the PBAF complex positively regulates ISGs. However, Dr. Wucherpfennig's group found that IFN α/γ responsive genes were significantly up-regulated in ARID2 and PBRM1-deficient B16F10 melanoma cells, leading to enhanced T cell infiltration and regression of murine melanoma (53). Recently, Dr. Wucherpfennig's group reported that CARM1 genetic knockout or inhibition in T cells activates type I interferon response in tumor cells (48). Our findings that ISGs are activated by CARM1i or in BAF155 ^R1064A/K^ cells indicate that me-BAF155 antagonizes the assembly of the BCL11A/PBAF complex that is required for ISG activation. Thus far, what determines SWI/SNF assembly as BAF or PBAF remains unclear. In a previous study of SWI/SNF assembly using biochemical analyses, BAF155, or the closely related protein BAF170, was shown to be involved in the initial step of SWI/SNF complex assembly to form the initial BAF core, which then associated with BAF60, BAF57, and BAF47 to form the BAF core (37). Subsequently, the BAF core associates with ARID1A/B to form the BAF complex, or with ARID2 to form the PBAF complex. Recently, the cryo-EM structures of the nucleosome-bound, mammalian partial SWI/SNF complex supports the essential role of BAF155/170 dimer as the scaffold of SWI/SNF (37,54). Despite the high sequence homology between BAF155 and BAF170, as well as their functional redundancy, BAF170 is not a substrate of CARM1, whereas BAF155 is methylated at a single site, R1064 (10). Notably, BAF170 is present, but BAF155 is absent in the structure of BAF (54). Moreover, BAF155 R1064 is not resolved in the human BAF structure (35), prohibiting prediction of the effect of R1064 methylation on complex assembly. Whether BAF155 methylation affects SWI/SNF complex assembly warrants further investigation. EZH2 and BAF155 have been reported to co-regulate tumor suppressor genes, and inhibiting BAF155 methylation could lead to displacement of BAF155 by EZH2 in ovarian cancer (55). Whether EZH2 is involved in regulating ISGs in TNBC remains unclear. Nevertheless, our data showed that inhibition of BAF155 methylation resulted in dissociation of HDAC1, and recruitment of BCL11A and PBAF to activated ISGs, implying that BAF155 methylation represents a therapeutic vulnerability for targeting the SWI/SNF complex in cancer treatment.

Collectively, our studies elucidate two mechanisms through which me-BAF155 drives cancer metastasis (Model in Figure 7E). On the one hand, me-BAF155, likely in the absence of the entire SWI/SNF complex, is essential for BRD4 chromatin association. Inhibition of BAF155 methylation leads to nearly complete dissociation of BRD4 from SEs, and thus inhibits expression of SE-addicted oncogenes. Compared to CARM1i, BETi regulates a greater number of genes in this study, and has profound side-effects in clinical trials. Thus, CARM1i may substitute JQ1 as a novel anti-cancer epigenetic drug. On the other hand, me-BAF155 suppresses ISG expression. Either BAF155 methyl-defective mutants or CARM1 inhibition results in recruitment of BCL11A/PBAF to activate ISGs, leading to increased tumor infiltration of CD8^+^ T cells and enhanced T cell-mediated killing (Model in Figure 7F).

Given that CARM1i exhibits low cytotoxicity, yet displays anti-migratory effects *in vitro*, and strong anti-tumor effects in various *in vivo* models, we envision that CARM1i can be exploited as an anti-cancer therapeutic agent alone, or in combination with other therapies (e.g., immunotherapy) for the treatment of metastatic cancer. Moreover, drugs selectively targeting CARM1-mediated BAF155 methylation may join the family of small molecule inhibitors targeting SWI/SNF complex assembly and function (56), and are expected to exhibit profound anti-cancer effects, while eliciting even fewer side-effects as compared to CARM1 inhibitors.

## DATA AVAILABILITY

All sequence data utilized for this study is available the Gene Expression Omnibus (PRJNA754771). The entire primary dataset of flow cytometry is available online at Dryad repository: https://doi.org/10.5061/dryad.xksn02vgq.

## Supplementary Material

gkab1122_Supplemental_Files

## Notes

Present address: Shengjie Zhang, The Cancer Hospital of the University of Chinese Academy of Sciences (Zhejiang Cancer Hospital), Institute of Basic Medicine and Cancer (IBMC), Chinese Academy of Sciences, Zhejiang 310022, China.
