# Supplementary material for: BAF155 methylation drives metastasis by hijacking super-enhancers and subverting anti-tumor immunity
Source: Nucleic Acids Res. 2021 Nov 19;49(21):12211–33. doi: 10.1093/nar/gkab1122 (PMC8643633; doi:10.1093/nar/gkab1122)
Supplement: gkab1122_Supplemental_Files [file gkab1122_supplemental_files.zip › 20211004 BAF155-BRD4_supl.pdf]

**Supplementary Figures**

**Figure S1 Me-BAF155 are enriched at super-enhancers (SEs) with BRD4/H3K27Ac/H3K4me1 ChIP-seq signals and BRD4 recruitment to SEs is dependent on BAF155 methylation in MDA-MB-468 cells**

A. Transcription factor motif analyses by HOMER at the shared regions between me-BAF155 and BRD4, among which the Top 10 ranked motifs are listed.

B. Knocking out BAF155 in MDA-MB-231 cells using CRISPR/cas9. DNA sequencing confirms homozygous -67 bp deletion in *BAF155* gene.

C. Real-time qPCR analyses of mRNA levels of metastasis inducers (CCL7, COL4A2, GADD45A and DDX18) or repressor genes (KISS1R, CDH11) in MDA-MB-231 BAF155 KO cells restored with $BAF155^{WT}$, $BAF155^{R1064K}$ or $BAF155^{R1064A}$. $^{**}p<0.01$; $^{*}p<0.05$

D. ChIP-qPCR analyses of me-BAF155 and BRD4 association with GADD45A, DDX18 and NDRG1 genes in MDA-MB-231 BAF155 KO cells or those restored with $BAF155^{WT}$, $BAF155^{R1064K}$ or $BAF155^{R1064A}$. $^{**}p<0.01$; $^{*}p<0.05$

**Figure S2 CARM1 inhibitors abrogate BRD4 recruitment to SEs and activation of oncogenes addicted to SEs in MDA-MB-468 cells.**

A. Real-time qPCR analyses of CDH1, CDCA7, COL1A2 and DDX18 by treatment of JQ1 or TP-064 in MDA-MB-231 cells. $^{**}p<0.01$; $^{*}p<0.05$

B. ChIP-qPCR analysis of me-BAF155 and BRD4 binding to the indicated genes under JQ1 or TP-064 treatment in MDA-MB-231 cells. **$p<0.01$; *$p<0.05$; NS: not significant

C. Genome browser snapshot of me-BAF155, BRD4, H3K27Ac ChIP-seq signals at *MYC* after treatment with vehicle (*black*), JQ1 (*red*) and TP-064 (*blue*) in MDA-MB-468 cells.

D. List of oncogenes whose putative SE-association were abrogated by both JQ1 and TP-064 (black) or individual agent (blue). SE-associated genes not affected by either agent were shown in red, and those contain new SEs after either drug treatment were shown in green.

E. Dose-dependent inhibition of BAF155 and PABP1 methylation by EZM2302.

F. Genome browser snapshot showing dissociation of BRD4 to *HIF1A* by EZM2302 treatment in MDA-MB-468 cells.

G. BRD4 ChIP-seq signals around 2kb of me-BAF155 vehicle peak summits with and without EZM2302 treatment in MDA-MB-468 cells.

H. Venn diagram showing the overlap of BRD4 dissociated ChIP-seq peaks upon treatment with TP-064 or EZM2302 in MDA-MB-468 cells.

I. Metagene plot showing dissociation of BRD4 binding peaks in TSS proximal regions (±2kb) by both JQ1 (red) and TP-064 (blue) as compared to vehicle control (black). On the contrary, TP-064 (blue), but not JQ1 (red), can dissociate me-BAF155 binding near TSS region in MDA-MB-468 cells.

J. Co-immunoprecipitation assays showing the decreased BRD4 association with me-BAF155 and BAF155 after treatment with ascending doses of TP-064 in MDA-MB-468 cells. BRD4 was detected in BAF155 or me-BAF155 immunoprecipitants by western blotting.

K. Nuclear BRD4 immunofluorescence signal after treatment with vehicle, TP-064, or 1,6-hexanediol in MDA-MB-468 cells (top) and quantification of BRD4 intensity normalized with vehicle control of 10 cells (bottom). ** $p<0.01$; Scale bars represent 10 $\mu$m.

**Figure S3 TP-064 exhibited anti-migratory effects in several TNBC cell lines**

A. Western blotting showing the time-dependent inhibition of endogenous BAF155 and PABP1 methylation by TP-064.

B. Percentage of mitotic cells harboring proper or improper chromosome segregation in JQ1 or TP-064 treated MDA-MB-468 cells.

C. Cell viability ratio representing the cell viability after treatment with ascending concentrations of JQ1, TP-064 or both normalized with vehicle control in SUM159 and SUM159R cells.

D. Representative images of migrated cells after treatment with ascending doses of JQ1, TP-064 or both in transwell assays for SUM159 (*top*) and SUM159R (*bottom*) cells.

E-F. JQ1 and TP-064 elicited synergistic anti-migratory effects in SUM159 and SUM159R cells. Synergy was calculated by combination index (CI) based on enzyme kinetic models of Chou-Talalay for drug combination of JQ1 X TP-064. CI plot was represented by fraction inhibited (x-axis) and CI value substituted by log10 (y-axis). For normalized isobologram plot, CI scores are plotted by D1/Dx1 and D2/Dx2, where Dx1 (JQ1) and Dx2 (TP-064) represent the doses of individual drug, causing fractional inhibition effect x, respectively.

**Figure S4 JQ1 and CARM1 inhibitors decreased SE-addicted oncogene expression and abrogated tumor growth and metastasis in TNBC PDX models**

A. Average body weights of mice remained stable during treatment with vehicle, JQ1, EZM2302 or JQ1+EZM2302 in PDX HCI-002 xenograft model.

B. Gene set enrichment analysis (GSEA) of RNA-seq data (n=3) from MDA-MB-468 cells treated with JQ1, TP-064 or JQ1 and TP-064 in combination on 125 SE-signature genes defined by BRD4/H3K27Ac ChIP-seq data.

C. Scheme of experimental design of JQ1 or EZM2302 treatment using PDX HCI-009 xenograft model.

D. Growth curves show the tumor volumes under each treatment condition normalized by the pre-treated tumor volumes of HCI-009. **$p<0.01$

E. Average body weight of mice during the duration of drug treatment in PDX HCI-009 model.

F. GSEA of RNA-seq data (n=3) from HCI-009 tumors treated with JQ1, EZM2302 or JQ1 + EZM2302 on 125 SE-signature genes.

G. Real-time qPCR analyses of selected SE-regulated oncogenes after treatment with vehicle, JQ1, EZM2302, or both in PDX HCI-009 tumors.

H. Western blotting of SE-regulated oncoproteins after treatment with indicated drugs in HCI-009 PDX.

I. Detection of lung micrometastasis by immunostaining of human-specific mitochondria antigen in lung tissues of HCI-009 PDX, where HCI-009 primary tumor was used as a positive control.

J. Micrometastasis cancer cells in lung tissues were quantified under each treatment condition in HCI-009 xenograft model. **p<0.01; *p<0.05; NS: not significant

**Figure S5 EZM2302 inhibited while JQ1 promoted 4T1.2 lung metastasis by regulating a variety of immune cells infiltrating to tumors.**

A. Scheme of experimental design testing the preventive effects of drugs on 4T1.2 tumor metastasis. Mice were pre-treated with vehicle, JQ1, EZM2302, or both three days prior to 4T1.2-luciferase cell implantation to mammary fat pads.

B. Growth curves of 4T1.2 primary tumors during one-month treatment with vehicle, JQ1, EZM2302, or both. **p<0.01

C. Luciferase signals of 4T1.2 tumor cells metastasize to lungs in the indicated treatment groups of the preventative scheme. *p<0.05

D. Bioluminescence imaging of 4T1.2-luciferase tumors in mammary fat pads and lungs after treatment with vehicle, JQ1, EZM2302, or both preventative scheme.

E. Flow cytometry analyses of CD4 and CD8 expressing cells from blood samples of 4T1.2 syngeneic mouse models under indicated treatment conditions on day 30.

F. Average body weights of all treatment groups remain unchanged during treatment with vehicle, two doses of anti-CD8 antibodies, EZM2302 and EZM2302 in combination with anti-CD8 antibodies in the 4T1.2-luciferase implantation model.

G. JQ1 treatment decreased Jurkat T cell numbers when co-cultured with MDA-MB-468 cells. **p<0.01; NS: not significant

H. TP-064 increased but JQ1 decreased Jurkat T cell cytotoxicity as measured by granzyme B activity when co-cultured with MDA-MB-468 cells for 48 hours. **p<0.01

**Figure S6 CARM1 inhibitor promotes immune response via activation of IFN α/γ pathway signaling in TNBCs.**

A. Venn diagram showing the overlap of DEGs induced by JQ1, EZM2302 or JQ1 + EZM2302 in 4T1.2 cells.

B. Venn diagram showing the overlap of activated or repressed DEGs induced by JQ1, CARM1 inhibitor, and combination of JQ1 with CARM1 inhibitor in MDA-MB-468, HCI-002 and 4T1.2 models.

C. Bar graph showing the relative DEGs in activated or repressed gene categories by treatment with JQ1, CARM1i or both in MDA-MB-468, HCI-002 and 4T1.2 models.

D. Hallmark gene sets enriched of activated (*red*) or repressed (*blue*) genes by JQ1 or EZM2302 treatment in HCI-002 and 4T1.2 models.

E. GSEA of RNA-seq data (n=3) from HCI-002 or 4T1.2 tumors treated with JQ1, EZM2302 or JQ1 and EZM2302 in combination on IFN α pathway related genes.

F. Heatmap of DEGs log2 (fold change) induced by JQ1, CARM1 inhibitor or both in MDA-MB-468 (*left*), HCI-002 (*middle*) and 4T1.2 (*right*).

G. Immunoblotting of putative transcription factors regulating IFN related proteins in response to JQ1, TP-064 or both treatment in MDA-MB-468 cells.

H. Q-RT-PCR analyses of mRNA levels of *BCL11A* after treatment with JQ1, TP-064 or both in MDA-MB-468 cells. **$p<0.01$

I. TP-064 treatment enhanced the formation of BCL11A and PABF complex revealed by co-IP. Immunoprecipitation was performed using BCL11A and BAF155 antibodies after treatment of MDA-MB-468 cells with JQ1, TP-064 or both. Indicated proteins in immunoprecipitants were detected by western blotting.

J. Western blotting showing the up-regulation of BCL11A in MDA-MB-231 BAF155 KO cells and those restored with $BAF155^{R1064K}$ and $BAF155^{R1064A}$, as compared to parental and $BAF155^{WT}$ restored MDA-MB-231 cells.

K. Real-time qPCR analyses of mRNA levels of BCL11A and IFN pathway genes in MDA-MB-231 BAF155 KO, $BAF155^{WT}$, $BAF155^{R1064K}$ and $BAF155^{R1064A}$ MDA-MB-231 cells. **$p<0.01$

**Figure S7 Optimization of me-BAF155 immunofluorescence staining in human CTC samples.**

A. Methodological overview outlining the processes from CTC enrichment of human blood sample to microscopic evaluation.

B. ExtractMax Exclusion-based Sample Preparation (ESP) technology achieves high yield CTC capture and manipulation with antibody-based magnetic movement using an automated magnetic arm on a pipetting robot (Gilson) and forces of surface tension to hold reagents in their respective wells (commercially available consumables). Technology schematic previously published (Guckenberger et al., Anal Chem, 2016).

C. Quantitative Microscopy is achieved by allowing cells to settle in a glass-bottomed chamber slide then imaging the entire chamber with a grid of 15x15 smaller image tiles of 20x magnification maintaining uniform focus with automated step-by-step focusing.

CTCs are identified with automated binary object algorithms (NIS Elements) as cells expressing pCK without exclusion channel markers, then objectively quantified for me-BAF155 nuclear expression.

D. Optimization of me-BAF155 staining by immunofluorescence in MDA-MB-468 cell line. The cells were stained and imaged using nuclear marker Hoechst, epithelial cell surface marker EpCAM (488 nm) and me-BAF155 (594 nm); scale bars represent 10 μm.

E. Nuclear immunostaining of me-BAF155 in EpCAM expressing MDA-MB-468 and MDA-MB-231 cells, but not in BAF155 KO MDA-MB-231 cells. BF denotes bright field. Scale bars represent 10 μm.

# Supplementary Figure 1

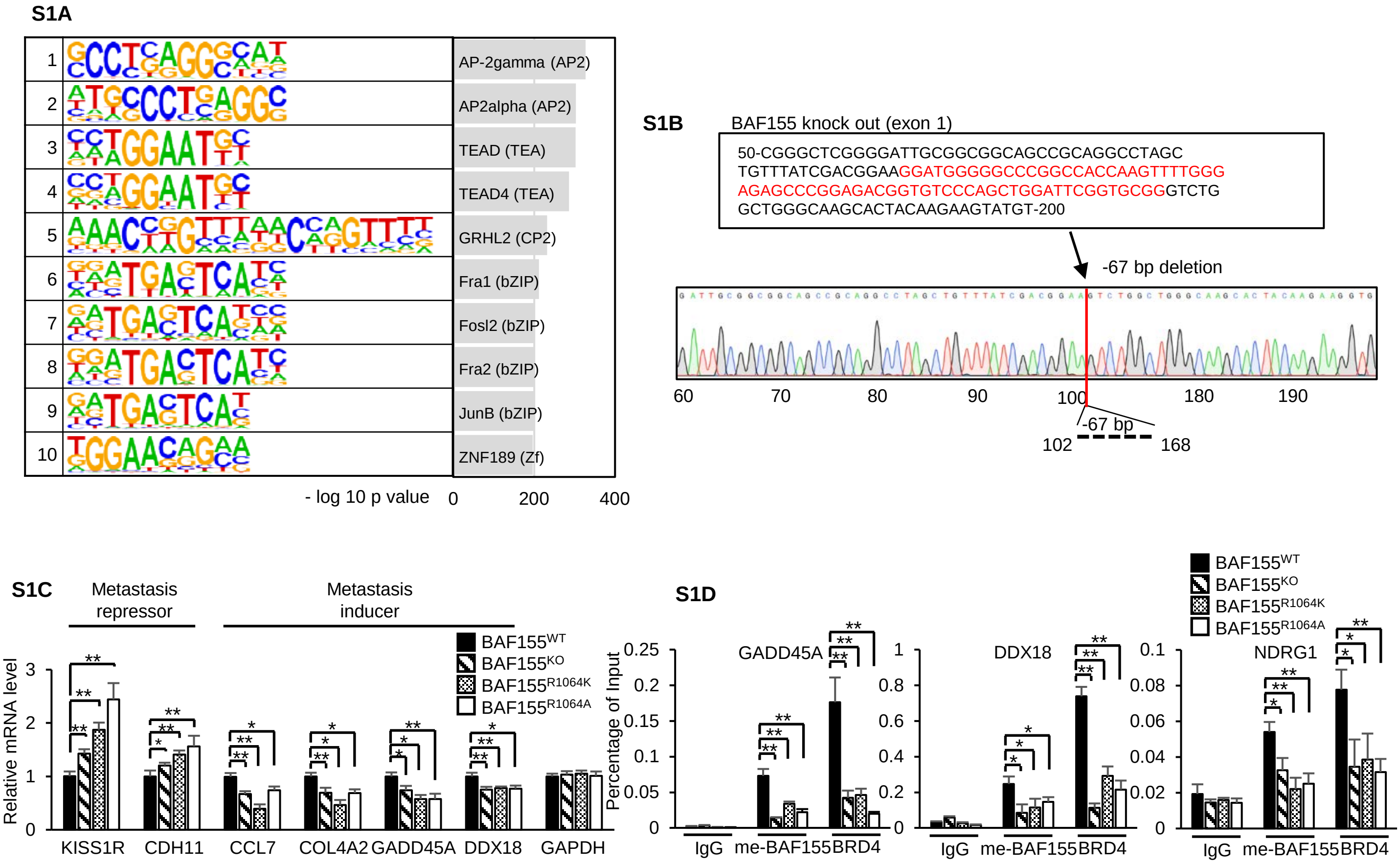

# Supplementary Figure 2

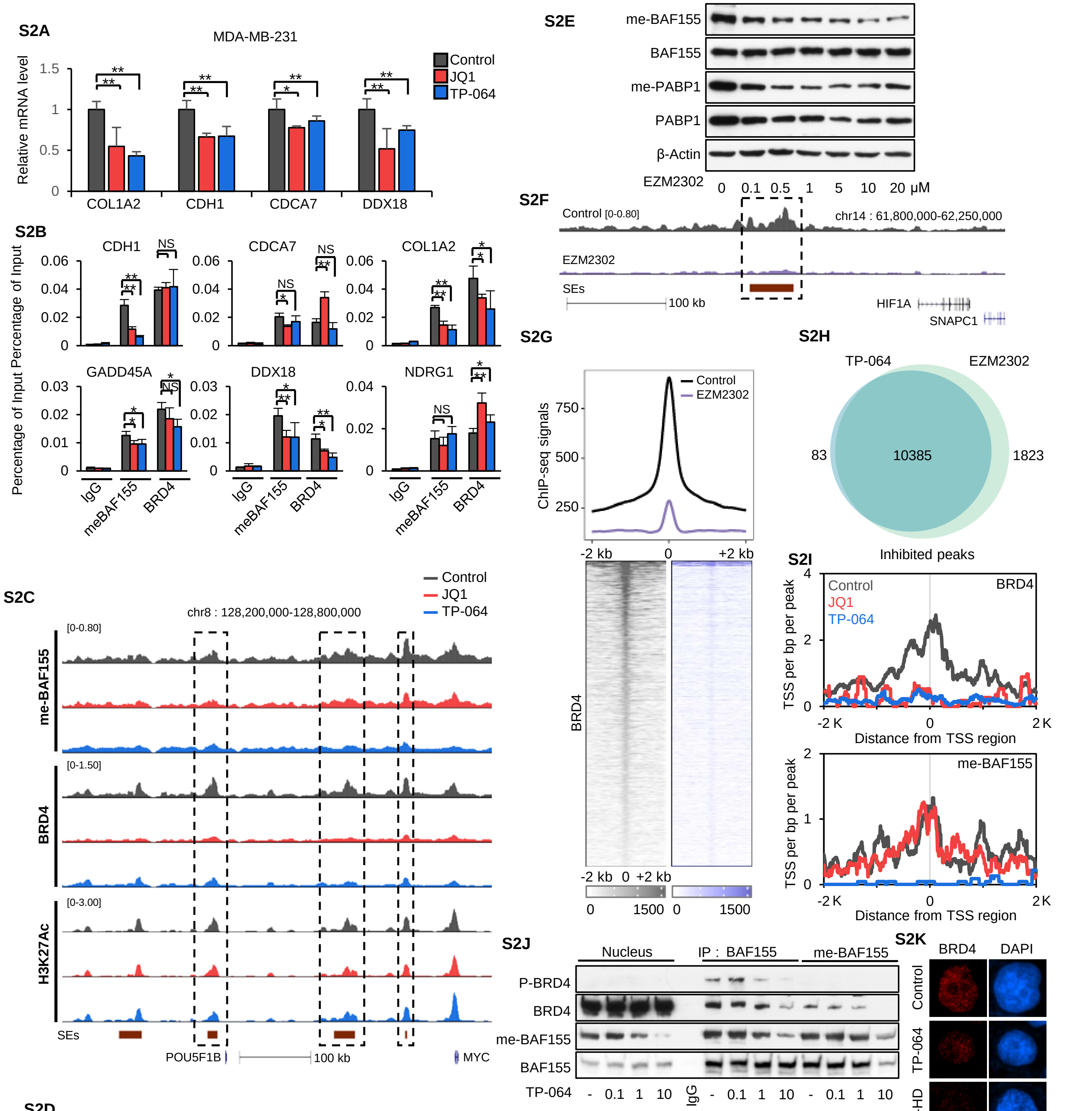


S2D

| | | | | | | | | | | | | | |
|---|---|---|---|---|---|---|---|---|---|---|---|---|---|
| ABCA4 | C21orf91 | CYP1B1 | FLJ33630 | IL22RA2 | LEPREL1 | MMP7 | OR1I1 | RNF144B | SYN3 | VPS13D | EHF | AHR | JQ1 |
| ADAM28 | C3orf57 | CYYR1 | FLJ41200 | IRF2BP2 | LOC100188947 | MOGAT1 | PDHX | SH3TC2 | TACSTD2 | ZMYND8 | VOPP1 | VSTM2A | |
| AK5 | C6orf223 | DDIT4 | FMO2 | IRX3 | LOC284751 | MTUS1 | PECAM1 | SHB | TFAP2A | ZNF608 | AMOTL2 | PREP | TP-064 |
| ANKH | C8orf75 | DIRC3 | FMO6P | ITGB6 | LOC727677 | MYC | PIK3R1 | SLC10A2 | TGIF1 | ZNF621 | EDN1 | RAD51L1 | |
| ARID1A | C9orf3 | DLGAP1 | GFOD1 | ITPR1 | LRIG2 | MYCNOS | PLK2 | SLC1A2 | TM4SF1 | ZNF673 | EIF3H | SLC1A3 | |
| ARID5B | CD40LG | DNAJC5B | GNE | KBTBD8 | MAML2 | NCAPH | POU2AF1 | SLC44A3 | TMEM123 | EGFR | FTO | SOX9 | |
| ASB7 | CD44 | DUSP4 | GPR98 | KCNIP1 | MFSD1 | NCRNA00111 | PRKCH | SLC45A1 | TMEM45A | SEC61G | GPR110 | TBL1XR1 | |
| ASB9P1 | CD59 | EFNA5 | HES1 | KCNN4 | MIMT1 | NCRNA00184 | RAB31 | SLC9A8 | TOMM20 | POU5F1B | LOC152225 | TGFB2 | |
| AZGP1 | CITED4 | ELF3 | HORMAD2 | KIF4B | MIR21 | NRIP1 | RAI14 | SLITRK6 | TPRG1 | | LOC642587 | TNS3 | |
| BBS12 | CLDN10 | ERRFI1 | HSD17B2 | KIRREL3 | MIR2278 | NRSN2 | RANBP3L | SNTB1 | TRPS1 | | MARCKS | LRRC4C | |
| BCL2L14 | CLDN23 | FAM176A | IER5 | KIT | MMADHC | NTN4 | RERG | SPP2 | TSC22D3 | | PHACTR1 | TPBG | |
| BHLHE40 | CMAH | FLJ22536 | IFNGR1 | KYNU | MMP20 | OLFM4 | RND3 | SUMO1P1 | TSPAN1 | | PKD1L1 | | |

Control

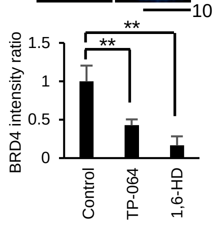


Control group only
Not changed by JQ1 or TP-064
Remained after JQ1 or TP-064
New site after JQ1 or TP-064

# Supplementary Figure 3

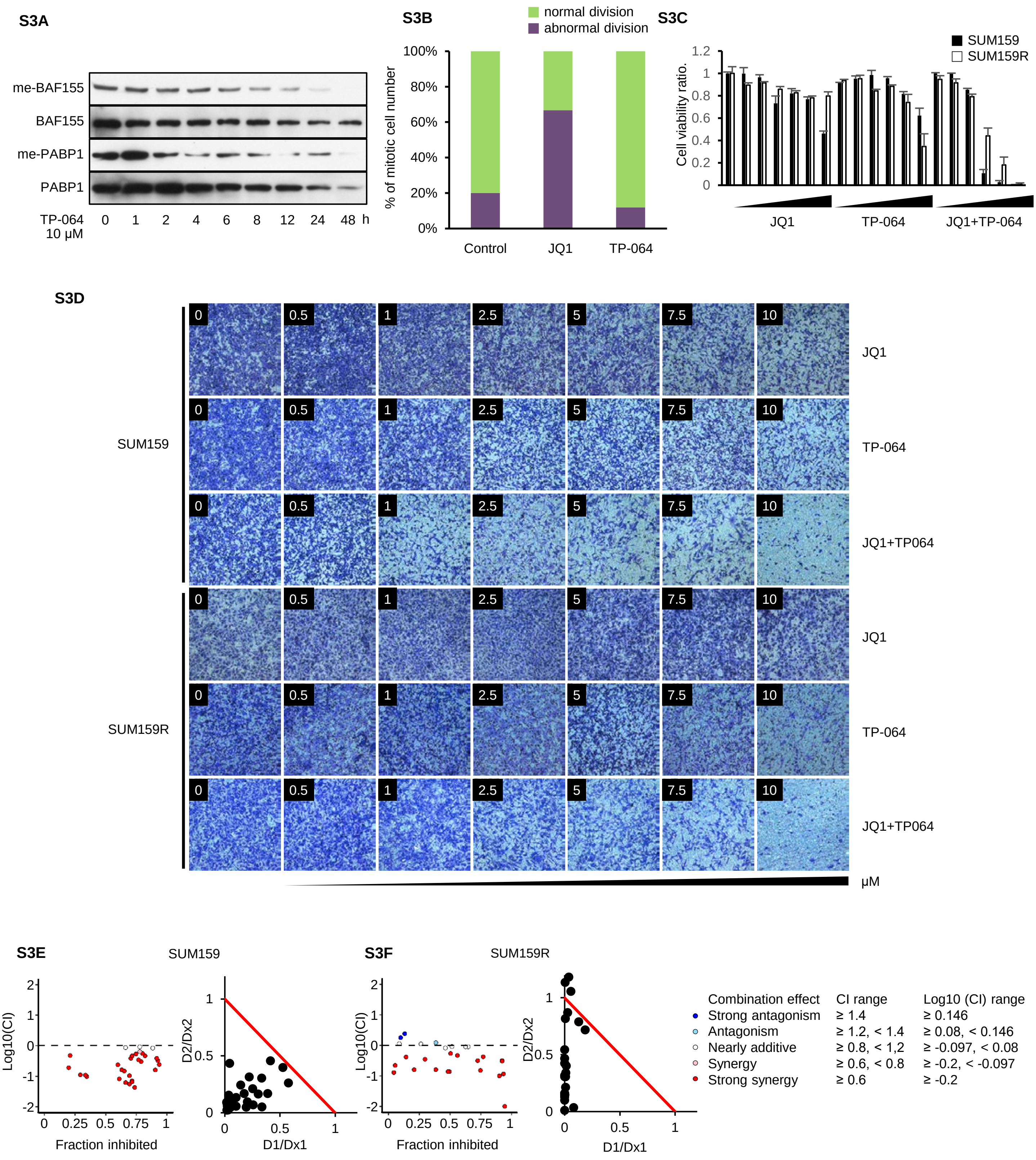

# Supplementary Figure 4

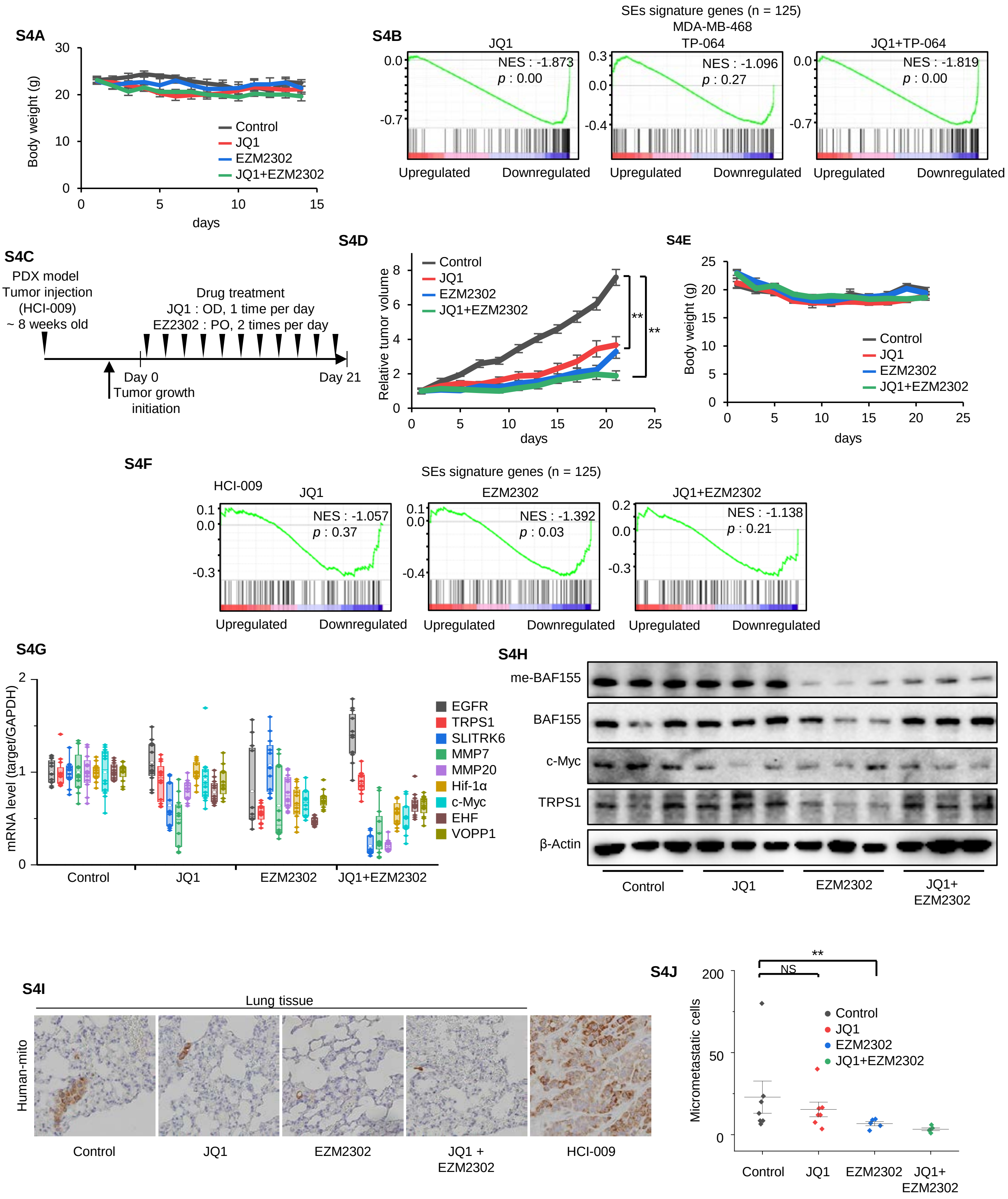

# Supplementary Figure 5

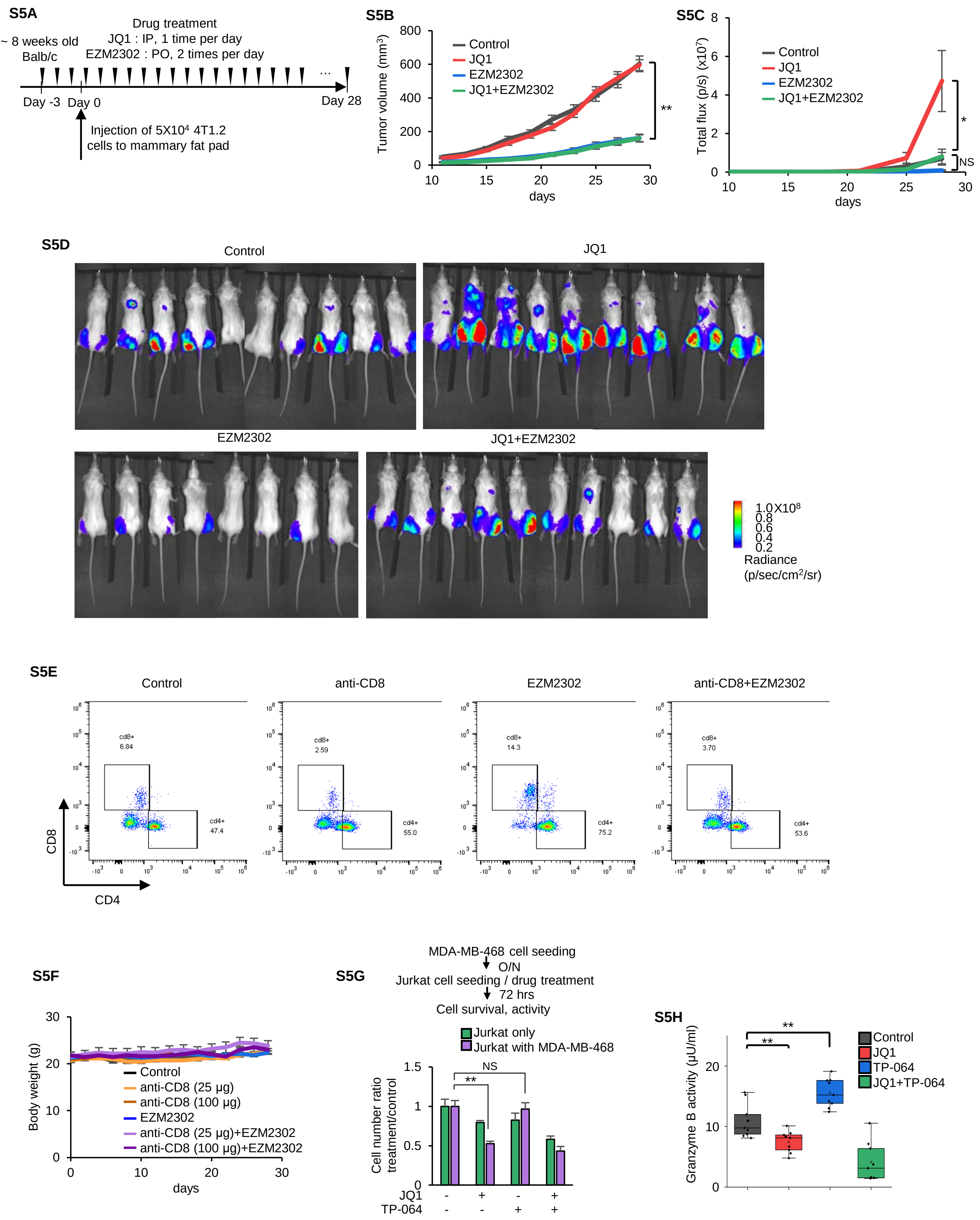

# Supplementary Figure 6

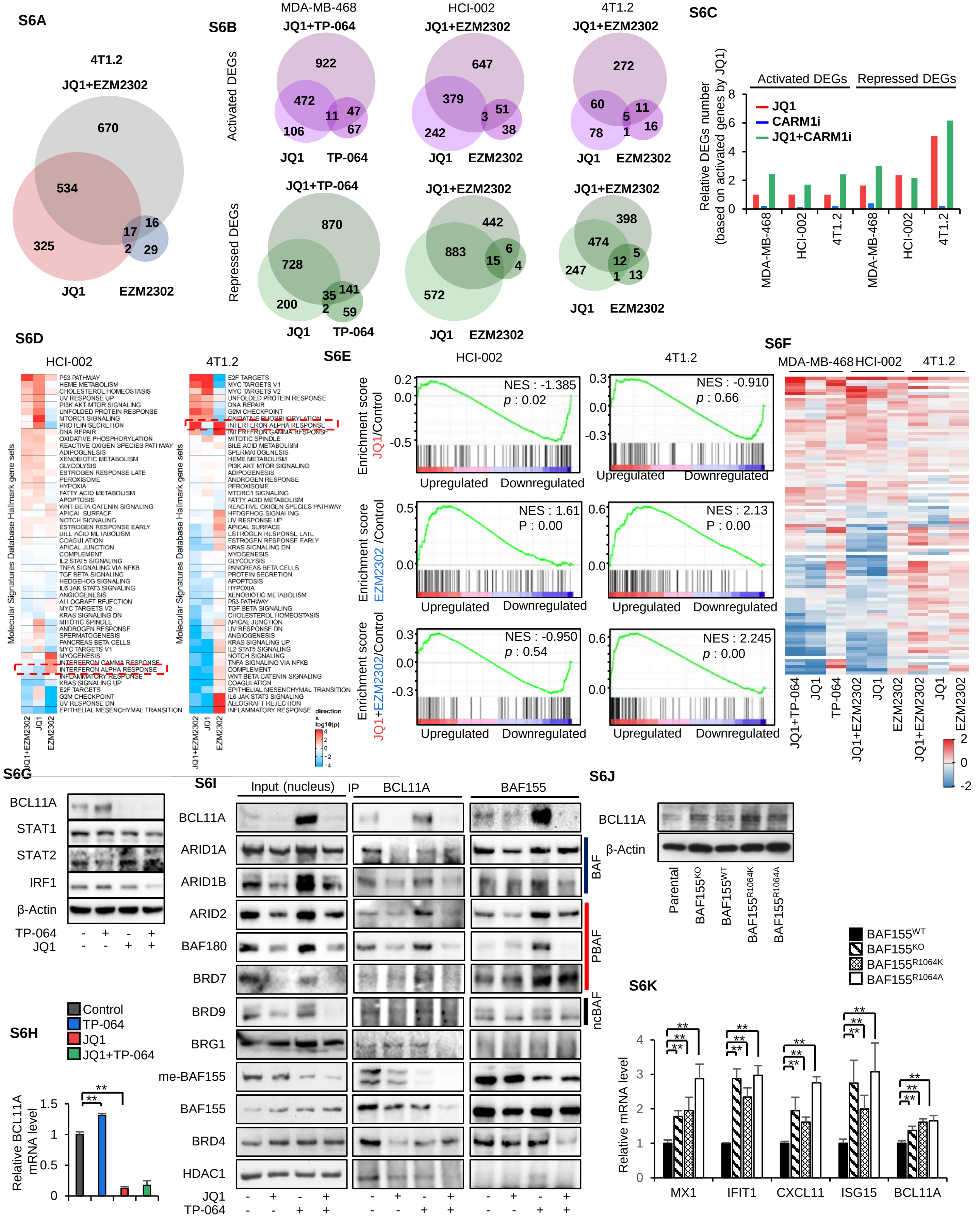

# Supplementary Figure 7

S7A Overview of Patient Sample CTC Isolation, Staining and Image Acquisition Workflow

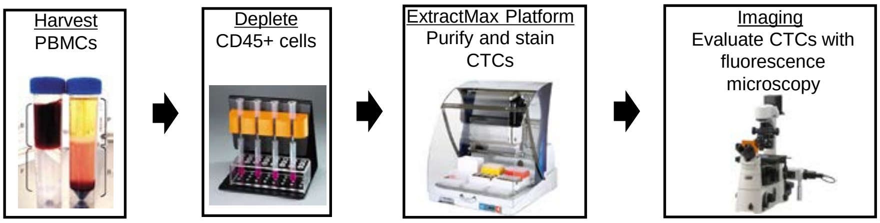


S7B. ExtractMax ESP Technology Overview

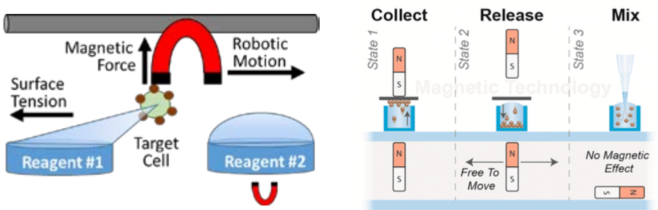


S7C. Quantitative Microscopy Workflow

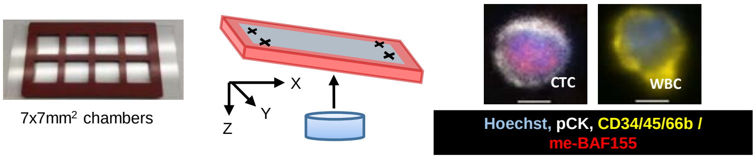


S7D

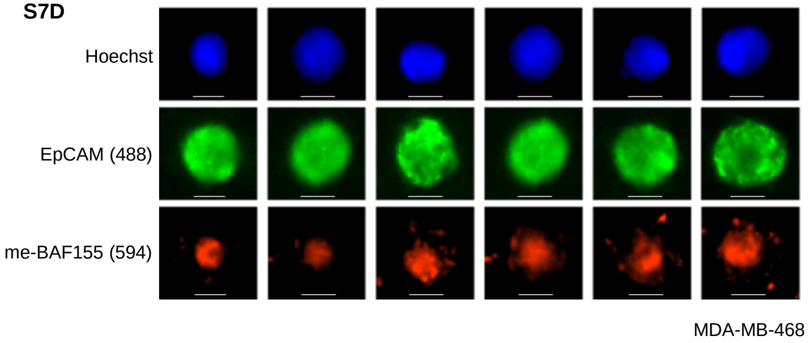


S7E

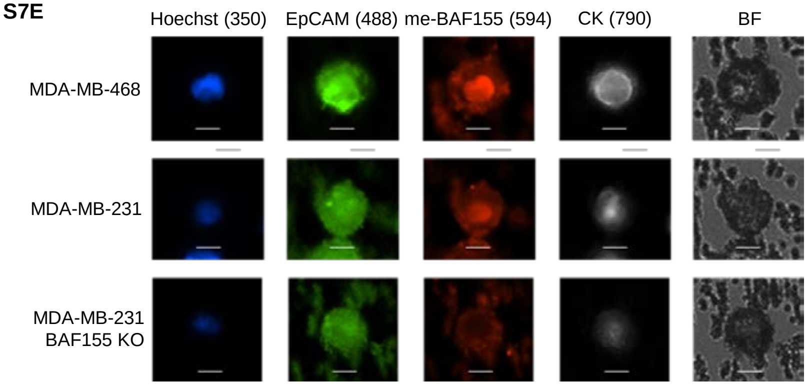

# Supplementary Table 1

| | | 455 | 493 | 12 | 453 | 464 | 548 | 552 |
|---|---|---|---|---|---|---|---|---|
| **ID** | | **455** | **493** | **12** | **453** | **464** | **548** | **552** |
| **Histology** | | Adenocarcinoma; lobular carcinoma | Carcinoma grade 3 | Ductal Carcinoma | Moderately to poorly differentiated carcinoma | Invasive ductal carcinoma | Ductal Carcinoma In Situ | Ductal Carcinoma |
| ER | | + | - | + | - | + | - | - |
| PR | | + | - | - | - | + | - | - |
| HER2 | | - | - | - | + | - | - | - |
| **Site of primary tumor** | | L | L | R | L | L | R | R |
| **Site(s) of metastases** | Bone | X | X | X | X | X | | |
| | Liver | | X | | X | | | X |
| | Brain | | | | X | | | |
| | Lymph nodes | | | | | | X | |
| | Contralateral breast | | | | | | | X |
| | Lung | | | | | | | X |
| | Adrenal | | | | | | | X |
| | Kidney | | | | | | | X |
| | Peritoneum | | | | | | | X |
| **Disease status** | | Stable | Responding | Stable | Stable | Stable | Stable | Stable |
| **Current therapy** | | exemestane, everolimus | Nab-paclitaxel | Eribulin | Trastuzumab, pertuzumab | Capecitabine, zometa | Eribulin | Carboplatin, gemcitabine |
| **Prior treatments** | Radiation | X | | X | X | X | | |
| | Letrozole | X | | X | | | | |
| | Palbociclib | X | | X | | | | |
| | Zometa | X | | | | | | |
| | Fubestrant | X | | | | | | |
| | Doxorubicin | | X | | | | | X |
| | Cyclophosphamide | | X | | | | | X |
| | Bicalutamide | | X | | | | X | |
| | Ribociclib | | X | | | | X | |
| | Carbo/gem | | X | X | | | | X |
| | Immunotherapy | | X | | | | | |
| | Nab-paclitaxel | | X | | | | | |
| | Mastectomy | | | X | | | | X |
| | Adriamycin | | | X | | | | |
| | Cytoxan | | | X | | | | |
| | Tamoxifen | | | X | | | | |
| | Anastrozole | | | X | | X | | |
| | Estradiol | | | X | | | | |
| | Capecitabine | | | X | | X | X | X |
| | Exemestane | | | X | | | | |
| | Abaraxane | | | X | | | | |
| | Taxol | | | | X | | | X |
| | Lumpectomy | | | | | | | X |
| | Faslodex | | | | | X | | |
| | Docetaxel-Cytoxan | | | | | | X | |
| | Eribulin | | | | | | X | |
| | Pembrolizumab | | | | | | X | |

**Supplementary Table 1 Clinical Details of the Patient Cohort.** Clinical details were tabulated for each patient evaluated for CTC me-BAF155 expression. Patient disease status at the time of draw was determined by clinician assessment of radiographic imaging results using RECIST criteria.
